# Interspecific Variation in One-Carbon Metabolism within the Ovarian Follicle, Oocyte, and Preimplantation Embryo: Consequences for Epigenetic Programming of DNA Methylation

**DOI:** 10.3390/ijms22041838

**Published:** 2021-02-12

**Authors:** Constance E. Clare, Valerie Pestinger, Wing Yee Kwong, Desmond A. R. Tutt, Juan Xu, Helen M. Byrne, David A. Barrett, Richard D. Emes, Kevin D. Sinclair

**Affiliations:** 1 Schools of Biosciences and Veterinary Medicine and Science, University of Nottingham, Sutton Bonington, Leicestershire LE12 5RD, UK; stxecc@exmail.nottingham.ac.uk (C.E.C.); valeriepestinger@icloud.com (V.P.); sbzwyk@exmail.nottingham.ac.uk (W.Y.K.); sbzdt2@exmail.nottingham.ac.uk (D.A.R.T.); juan.xu918@gmail.com (J.X.); richard.emes@nottingham.ac.uk (R.D.E.); 2 Centre for Analytical Bioscience, School of Pharmacy, University of Nottingham, Nottingham NG7 2RD, UK; david.barrett@nottinbgham.ac.uk; 3 Mathematical Institute, University of Oxford, Woodstock Road, Oxford OX2 6GG, UK; helen.byrne@keble.ox.ac.uk

**Keywords:** one-carbon metabolism, methionine, Assisted Reproduction Technologies (ART), embryo, Epigenetics, DNA methylation, genomic imprinting

## Abstract

One-carbon (1C) metabolism provides methyl groups for the synthesis and/or methylation of purines and pyrimidines, biogenic amines, proteins, and phospholipids. Our understanding of how 1C pathways operate, however, pertains mostly to the (rat) liver. Here we report that transcripts for all bar two genes (i.e., *BHMT*, *MAT1A*) encoding enzymes in the linked methionine-folate cycles are expressed in all cell types within the ovarian follicle, oocyte, and blastocyst in the cow, sheep, and pig; as well as in rat granulosa cells (GCs) and human KGN cells (a granulosa-like tumor cell line). Betaine-homocysteine methyltransferase (BHMT) protein was absent in bovine theca and GCs, as was activity of this enzyme in GCs. Mathematical modeling predicted that absence of this enzyme would lead to more volatile S-adenosylmethionine-mediated transmethylation in response to 1C substrate (e.g., methionine) or cofactor provision. We tested the sensitivity of bovine GCs to reduced methionine (from 50 to 10 µM) and observed a diminished flux of 1C units through the methionine cycle. We then used reduced-representation bisulfite sequencing to demonstrate that this reduction in methionine during bovine embryo culture leads to genome-wide alterations to DNA methylation in >1600 genes, including a cohort of imprinted genes linked to an abnormal fetal-overgrowth phenotype. Bovine ovarian and embryonic cells are acutely sensitive to methionine, but further experimentation is required to determine the significance of interspecific variation in BHMT expression.

## 1. Introduction

One-carbon (1C) metabolism refers to a series of interlinking and closely associated metabolic pathways that serve to provide methyl groups (1C units) for the de novo synthesis and/or methylation of purines and pyrimidines, biogenic amines, proteins, and phospholipids; all of which are critical for cellular function [1,2]. These cellular processes are particularly important during the periconceptional period, which witnesses sweeping epigenetic alterations to DNA and associated proteins during the latter stages of gametogenesis and early embryogenesis [3]. These epigenetic modifications initially enable the two terminally differentiated gametes to form a totipotent zygote upon syngamy and, subsequently, to drive the processes of lineage specification, cellular differentiation and organogenesis during the first few weeks of pregnancy. A number of studies across a diverse range of species, including humans, have demonstrated that disturbances to 1C metabolism brought about by parental malnutrition [4,5,6] and/or exposure to environmental chemicals [7,8] during the periconceptional period, or arising as a consequence of assisted reproduction (ART) [9,10,11], can lead to epigenetic alterations to chromatin with long-term consequences for offspring health and wellbeing.

**Figure 1:**
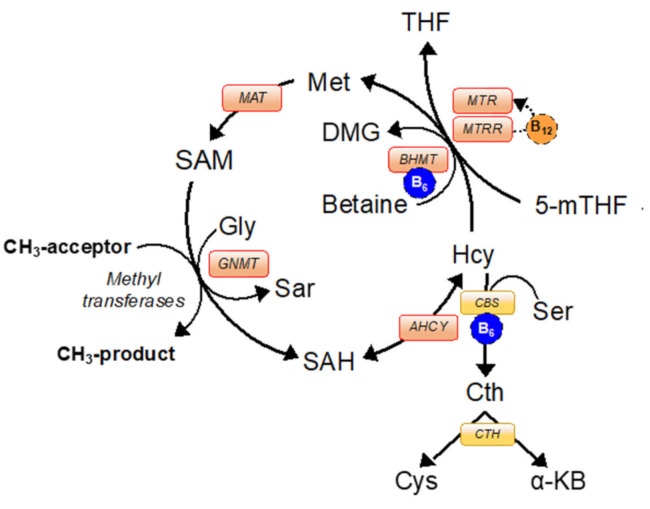
The methionine cycle facilitates re-methylation of homocysteine to methionine via methionine synthase (MTR) and betaine-homocysteine S-methyltransferase (BHMT) enzymes. Methionine cycle enzymes (red boxes): AHCY; S-adenosyl-L-homocysteine hydrolase; BHMT, betaine-homocysteine S-methyltransferase; MTR, methionine synthase; MTRR, methionine synthase reductase; MATI/II/III, methionine adenosyltransferase; GNMT, glycine N-methyltransferase. Transsulfuration pathway enzymes (yellow boxes): CBS, cystathionine β-synthase; CTH, cystathionine γ-lyase. Enzyme cofactors: B6 (blue circle) and B12 (orange circle). Substrates: 5-mTHF, 5-methyltetrahydrofolate; Cth, cystathionine; Cys, cysteine; DMG, dimethylglycine; Gly, glycine; Hcy, homocysteine; Met, methionine; SAH, S-adenosylhomocysteine; SAM, S-adenosylmethionine; Sar, sarcosine; Ser, serine; THF, tetrahydrofolate; α-KB, α-ketobutyrate.

Much of our understanding of how 1C pathways operate within cellular metabolism pertains to studies undertaken in the liver, particularly in the rat liver on which most mathematical models are based [12,13], with comparatively little known about how these cycles operate within reproductive tissues. We reported that transcripts for specific enzymes, most notably betaine-homocysteine methyltransferase (BHMT; EC 2.1.1.5), were not expressed in any of the somatic cell types found within the ovarian follicle, oocyte, and preimplantation embryo in the cow [14]. Given its role in the re-methylation of homocysteine (Hcy) to methionine [2,15] (Figure 1), we hypothesized that lack of expression of BHMT would render the oocyte and preimplantation embryo particularly sensitive to fluctuations in 1C substrates/cofactors, such as methionine, folate, and vitamin B_12_ within parental diet and/or embryo culture media. Indeed, preliminary mathematical simulations using the model developed by Reed et al. [12] support this contention (Figure S1).

These observations, however, are not in complete agreement with those of Ikeda et al. [16], who reported transient expression of BHMT in cultured bovine embryos. They also appear to be at odds with data emerging from the mouse, which indicate the presence of an active BHMT-mediated transmethylation pathway operating within the blastocyst of this species [17,18]. In an attempt to reconcile these apparent discrepancies, we now report on studies that determined the expression of BHMT and other key 1C metabolism enzymes for different somatic cell types within the ovarian follicle of the cow, sheep, pig, rat, and human. These studies were extended to include transcript expression of key 1C enzymes in the oocyte and blastocyst of the cow, sheep and pig; BHMT activity was also determined in primary granulosa cells from both the cow and rat.

The study of Zhang et al. [18] reported that DNA methylation (detected as 5-methylcytosine (5mC) immunofluorescence) was decreased when both BHMT and folate-cycle activity were simultaneously inhibited in the mouse blastocyst; an effect that was reversed with the addition of supra-physiological (200 µM) concentrations of either methionine or S-adenosylmethionine (SAM) to culture media. However, a similar immunofluorescence approach with bovine blastocysts failed to report an effect of lower (0 to 21 µM) methionine concentrations within culture media on DNA methylation [19]. Immunofluorescence labelling of 5mC, whilst a valid methodology, lacks the sensitivity and base-pair resolution that sequencing based approaches have to offer; and this could explain the failure to detect an effect of methionine in this latter study with cultured bovine embryos. Immunofluorescence approaches also provide no information on the DNA sequences altered and gene networks affected. In contrast, using a micro-array-based platform, the inclusion of physiologically high (2 μM) doses of SAM during bovine embryo culture led to genome-wide hypermethylation, mainly in exonic regions and cytosine-phosphate-guanine dinucleotide (CpG) islands, with gene-pathway enrichment analysis pointing to alterations in base-excision repair and embryonic stem-cell pluripotency pathways [20]. 

With the foregoing discussion in mind, and following a cross-species comparison of 1C enzyme expression and activity within the ovarian follicle, oocyte and preimplantation embryo, this article presents data on the effects of altering methionine concentration (within physiological limits) during bovine in vitro embryo production (IVP) on epigenetic modifications to DNA methylation as determined by reduced representation bisulfite sequencing (RRBS). This technique was selected as it offers an established, well characterized, and efficient high-throughput method for establishing genome-wide methylation profiles with single base-pair resolution [21,22,23]. This phase of the study focused on the effects of altering methionine concentrations for three reasons. Firstly, methionine occupies a key position in 1C metabolism, being the immediate precursor molecule for SAM, a universal donor of methyl (1C) groups involved in a number of biochemical reactions including DNA methylation (Figure 1). Secondly, methyl donation for these reactions from folate (5-methyltetrahydrofolate) and/or from betaine (trimethylglycine) transits via methionine, which is also an essential amino acid of dietary origin and is often incorporated into embryo culture media. Thirdly, methionine inclusion levels in commercially available oocyte maturation and embryo culture media vary markedly between studies, ranging from 0 to 500 μM (Table S1) [24,25], and so often bear little relationship to levels found in biological fluids (19 to 45 μM) [26]. 

It follows that the formulation of appropriate and safe embryo culture media requires that the effects of varying levels of this key 1C substrate be assessed. To that end, we report that modest and physiologically relevant reductions in added methionine (from 50 to 10 µM) during bovine embryo culture leads to genome-wide alterations to DNA methylation in >1600 genes, including a cohort of imprinted genes involved in fetal development. We also confirm the lack of BHMT expression and enzyme activity in the bovine ovarian follicle. We propose that this may contribute to heightened sensitivity of ovarian and embryonic cells to fluctuations in 1C metabolism in this species. Although awaiting confirmation, this study indicates that interspecific variation in BHMT expression, in both somatic and embryonic lineages, may heighten sensitivity to altered 1C metabolism during the periconceptional period. 

## 2. Results

### 2.1. Expression of 1C metabolism Genes Differs between Species and Cell Type within Species

As a first step in establishing the extent to which 1C metabolic pathways operate within the ovarian follicle, the oocyte and preimplantation embryo, transcript expression for fourteen 1C related genes, representing the linked methionine, folate, and transsulfuration pathways [2], was determined in theca, granulosa, and cumulus cells, oocytes, and expanded blastocysts from the cow, sheep, and pig by quantitative real-time PCR (qPCR) (Table S2a). In these analyses, purity of granulosa and theca cell separation was confirmed by qPCR for two cell-specific steroidogenic enzymes (Figure S2). Transcript expression was also determined in rat primary granulosa cells and cultured KGN cells (a granulosa-like tumor cell line [27], which served as a surrogate for primary human granulosa cells. Liver served as a positive control for each species apart for humans, where cultured human hepatocellular liver carcinoma cell line (HepG2) cells [28] served as a surrogate. Although quantification was based on normalization to a single reference gene (*ACTB*), which we previously had deemed stable in the different cell types studied [29,30], it is important to note that establishing transcript presence, rather than transcript abundance, was the primary goal of this phase of the study. We report that transcripts for all but three of the 1C genes studied were expressed in all cell types and across all species (data not presented). In contrast, expression of transcripts for *BHMT*, *MAT1A*, and *MAT2A* (Figure 1) varied between cell type and between species. 

**Figure 2:**
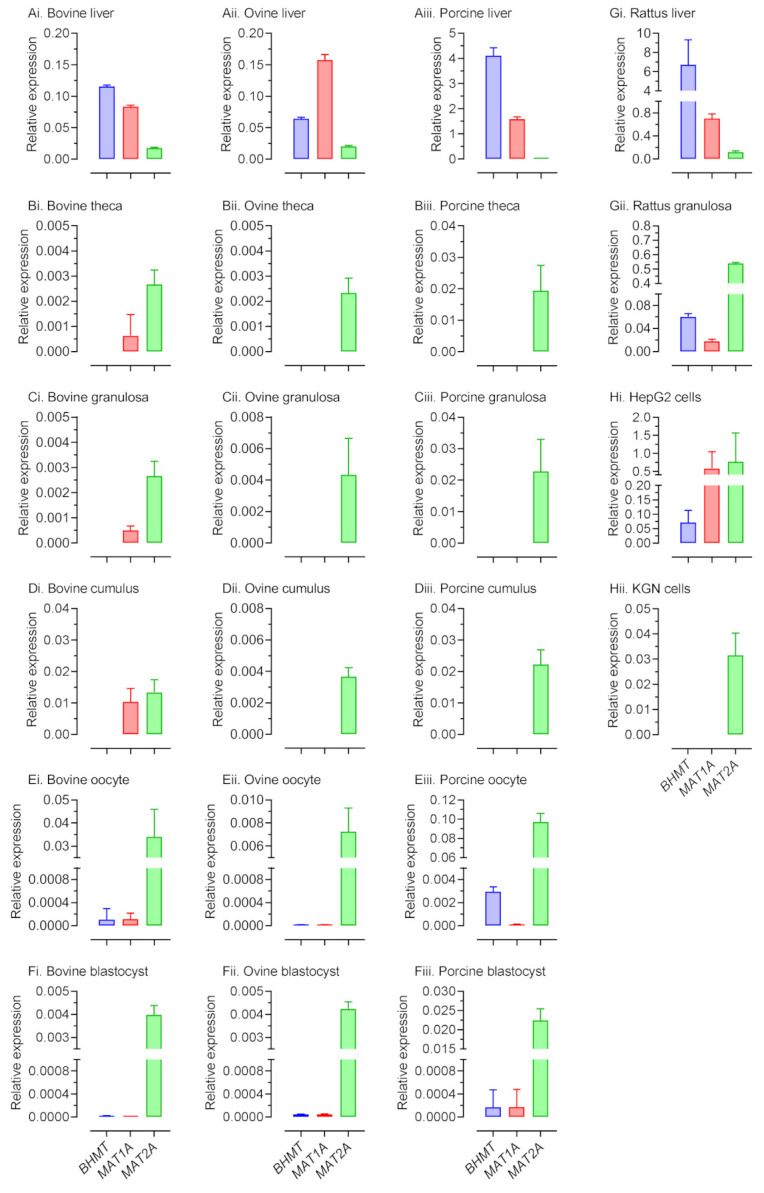
Relative (to *ACTB*) expression of transcripts for three one-carbon (1C)-genes in the liver (**A**i, ii, iii) and different somatic cell types of the ovarian follicle (theca, **B**i, ii, iii; granulosa **C**i, ii, iii; **D**i, ii, iii), the oocyte (**E**i, ii, iii), and blastocyst (**F**i, ii, iii) from three domestic animal species, together with transcript expression in rat granulosa cells (**G**ii) and liver (**G**i), and in two human cell lines (i.e., KGN (a granulosa-like tumor cell line; **H**ii) and human hepatocellular liver carcinoma cell line (HepG2) cells; **H**i) representing granulosa cells and liver, respectively.

Consistent with the study of Kwong et al. [14], transcripts for *BHMT*, whilst abundantly expressed in the bovine liver, were absent in all somatic-cell lineages of the ovarian follicle. A similar picture was evident in the sheep and pig. However, low abundance transcripts for this gene were detected in the bovine and porcine oocyte, and in the porcine blastocyst (Figure 2Ei,Eiii,Fiii). These latter observations are broadly in keeping with those of Ikeda et al. [16] and Jia et al. [31] in the cow and pig respectively. Likewise, *BHMT* expression was detected in rat granulosa cells (Figure 2Gii) but was absent in the equivalent human cell type (KGN cells; Figure 2Hii). Transcripts for *MAT1A*, expressed primarily in hepatocytes [32], were not detected in ovarian cells from the sheep and pig, nor in human (KGN) cells (Figure 2). However, they were detected in equivalent cells from both the cow and rat, as well as in the porcine blastocyst. The latter observation is consistent with that of Dalto et al. [33]. In contrast, transcripts for *MAT2A*, the enzyme product of which has a comparatively low Km for methionine, were detected in all cell types in all species studied. 

### 2.2. BHMT Expression and Activity within Ovarian Somatic Cells Differs between Species

**Figure 3:**
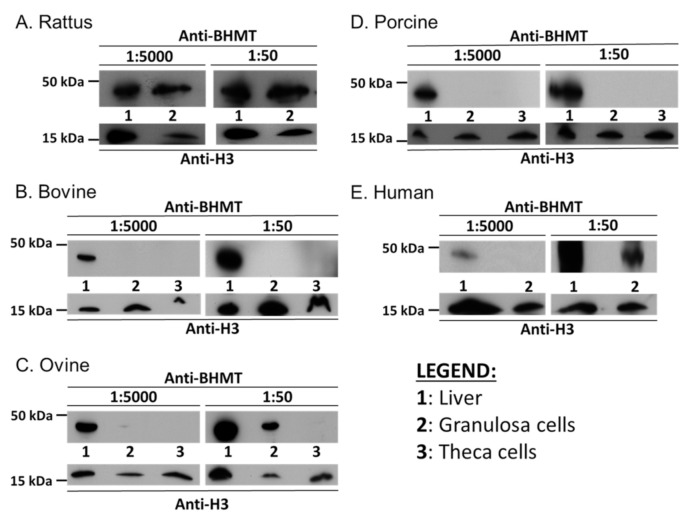
Expression of BHMT protein in primary cells from liver and granulosa of rat (**A**); liver, granulosa, and theca cells cow (**B**), sheep (**C**), and pig (**D**); as well as in human HepG2 and KGN cells (**E**). Moreover, 30 µg HepG2, granulosa, and theca cell lysate, and 10 µg of liver lysate, were loaded. Probing of membranes with anti-H3 served as a positive control. Antibody concentrations provided in Table S7.

**Figure 4:**
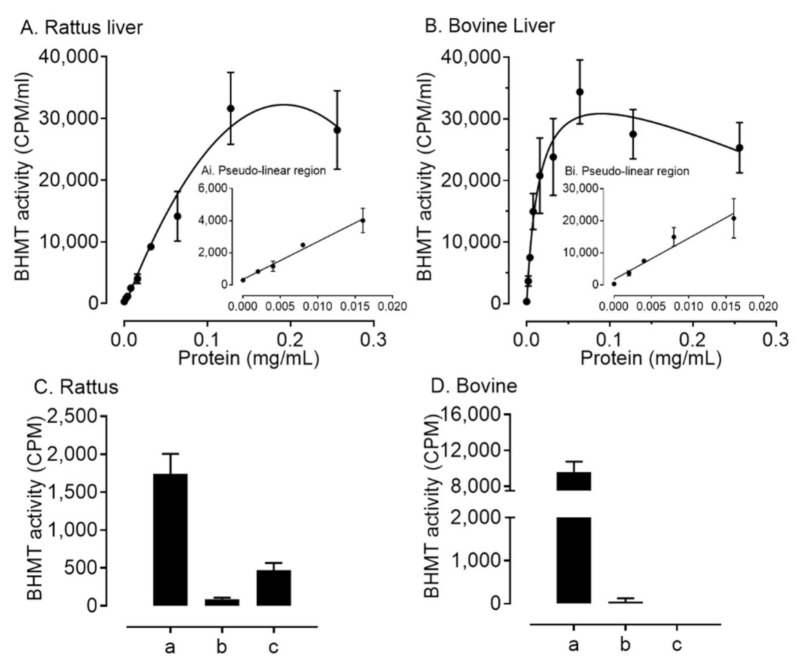
Validation of a BHMT assay in (**A**) rat and (**B**) bovine liver (means ± SEM; *n* = 3), and BHMT activity in (**C**) rat and (**D**) bovine granulosa cells. Activity is indicated as counts per minute (CPM) of ^3^H eluted from the column per mL, representing the total of BHMT products (i.e., methionine and dimethylglycine) produced. Means (± SEM; *n* = 3) were calculated for 10 mL HCl used for elution. Insets show the pseudolinear region on an expanded scale with the line fitted by linear regression (R^2^ = 0.989 in rat and R^2^ = 0.951 in bovine liver). Total liver protein (**a**, 0.008 mg/mL protein) for each species served as a positive control. Granulosa-cell protein for each species was loaded at two concentrations (**b**, 0.008 mg/L; **c**, 0.2 mg/mL).

We next established if the absence of transcripts for *BHMT* in the somatic-cell lineages of the ovarian follicle in the cow, sheep, pig, and human was consistent with an absence of BHMT protein. This was confirmed by Western blotting in both granulosa and theca cells, for the cow and pig, but very low-level expression was detected in sheep granulosa cells and human KGN cells (Figure 3). In contrast, and consistent with transcript expression, BHMT protein was detected in abundance in rat granulosa cells. Finally, in order to confirm that absence of both transcript and protein expression for BHMT in bovine granulosa cells was commensurate with a lack of enzyme activity, assays were performed to quantify the generation of BHMT products (methionine and dimethylglycine) from titrated betaine. Relative to enzyme activity detected in liver–protein lysates from both species, only low-level activity was detected in rat granulosa cells, and no activity was detected in bovine granulosa cells (Figure 4).

### 2.3. MAT Expression and Activity is Sensitive to Physiological Levels of Methionine during Bovine Granulosa-Cell Culture

**Figure 5:**
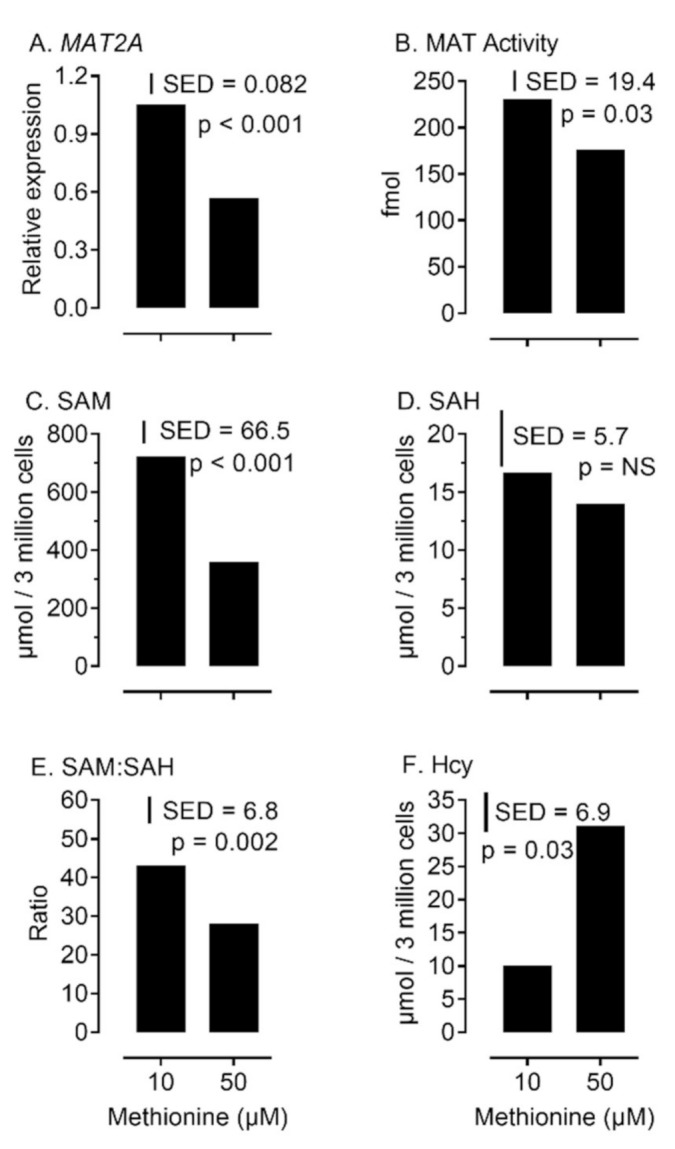
Effect of methionine concentration on methionine adenosyl-transferase 2a (*MAT2A*) transcript expression (**A**), MAT enzyme activity (**B**), and intra-cellular concentrations of 1C metabolites (S-adenosyl-methionine (SAM) **C**; S-adenosyl-homocysteine (SAH) (**D**); SAM:SAH ratio (**E**); and homocysteine (Hcy) (**F**)) in cultured bovine primary granulosa cells.

To gain an insight into the sensitivity of the ovary to varying concentrations of methionine, within physiological limits [26], bovine granulosa cells were cultured in the presence of either 10 or 50 µM added methionine. Both *MAT2A* expression (*p* < 0.001) and MAT enzyme activity (*p* = 0.03) increased at the lower concentration of methionine (Figure 5A,B). This was related to an increase (*p* < 0.001) in intracellular SAM, but not SAH, concentration (Figure 5C,D), and an increase (*p* = 0.002) in the ratio of SAM:SAH (Figure 5E). In contrast, intracellular Hcy concentration was lower (*p* = 0.03) at 10 relative to 50 µM added methionine (Figure 5F). Cellular egress of Hcy into surrounding culture media was also reduced at 10 compared to 50 µM added methionine (0.192 vs. 0.475 µmol/L/10^5^ cells; SED = 0.0924). Collectively these observations for Hcy are consistent with a reduced flux of methyl groups through this cycle at the lower concentration. 

### 2.4. Bovine In Vitro Embryo Production (IVP) is Sensitive to Physiological Concentrations of Methionine

Having established that bovine granulosa cells were responsive metabolically to added methionine concentrations within the range of 10 and 50 µM, we next established if altering the concentration of this essential amino acid within these physiological limits had an effect on embryo development. Methionine concentration was adjusted accordingly during IVP embracing in vitro oocyte maturation (IVM), fertilization (IVF) and embryo culture (IVC) (Figure S3) to reflect these levels in a study that involved maturing 2,558 cumulus–oocyte complexes (COCs) in 19 replicated experiments. The effect of reducing added methionine concentration from 50 to 10 µM led to modest, but statistically significant, reductions in the proportion of Day 8 (*p* = 0.015), particularly advanced Day 8 (*p* = 0.017), blastocysts. This was further reflected by a reduction (*p* = 0.006) in total cell number within Day 8 blastocysts (Table 1). 

### 2.5. Methionine Concentration Affects Global DNA Methylation during Bovine IVP

We next established the effects of altering methionine concentrations during IVM, IVF, and IVC on DNA methylation in Day 8 blastocysts. These embryos were immunodissected individually to separate inner-cell mass (ICM) and trophectoderm (TE) lineages (Figure S4), and DNA extracted from three replicate pools of ICM and TE cells, each derived from five stage-matched expanded blastocysts. RRBS (minimum difference of >20%) revealed that, relative to 50 µM methionine, IVP in the presence of 10 µM added methionine led to global hypomethylation in both cell lineages (Table 2A). However, the effect of reducing methionine concentration, on both the number and percentage of hypomethylated CpGs, was greater in the TE than in the ICM lineage. The number of differentially methylated CpGs between these two lineages was less at the lower methionine concentration (Table 2B). The lower concentration of methionine also increased the percentage of hypomethylated sites in the TE compared to the ICM lineage. The distribution of differentially methylated CpGs across genomic regions (promoters, intronic, exonic, CpG islands and CpG island shores) were similar between lineages and between methionine concentrations. Approximately 6–7% were located in gene promoters, 30–32% in exons, 62–64% in introns, 54% in CpG islands, and 46% in CpG island shores.

Gene set enrichment analysis (GSEA) revealed gene ontology (GO) terms and Kyoto Encyclopedia of Genes and Genomes (KEGG) pathways that were significantly enriched (False Discovery Rate (FDR) <0.05) with differentially methylated genes between the two physiological methionine concentrations (~10 v ~50 µM) during IVM and IVC. A total of 121 GO terms and 30 KEGG pathways containing genes with clusters of differentially methylated CpGs (≥5 differentially methylated CpGs within 1 kb) were enriched within the ICM, and 144 GO terms and 96 KEGG pathways containing genes with clusters of differentially methylated CpGs were enriched within the TE. As GSEA makes unrealistic assumptions about statistical independence and ignores the fact that some genes are co-expressed [34], many statistically significant pathways comprise a small number of differentially methylated genes of interest (GOI). Thus, in order to avoid testing overly narrow categories, the present study analyzed the top five GO terms and KEGG pathways that comprised the highest number of differentially methylated GOI (Table S3). Pathways with the greatest number of differentially methylated GOI that were enriched in the ICM were broadly associated with protein catabolism and autophagy. The term ‘cytosol’ represents 40 GOI involved in protein complex formation, whereas ‘hydrolase’ (18 GOI) and ‘proteolysis’ (11 GOI) terms relate to the hydrolysis of proteins into amino acids by the cleavage of peptide bonds. In the TE, ‘protein kinase activity’ comprised the greatest number (*n* = 15) of differentially methylated GOI. In addition, KEGG ‘pathways in cancer’ (13 GOI) were enriched in TE cells. Terms and pathways enriched in both cell lineages that had the greatest number of differentially methylated GOI were ‘metabolic pathways’, ‘phosphorylation’, and ‘kinase activity’. 

Genes with the greatest number of differentially methylated CpGs were selected from the first GO term and KEGG pathway to illustrate the effects of reducing methionine within physiological levels during bovine embryo culture (Table S4). At 131 and 146 differentially methylated CpGs in the ICM and TE, respectively, dopamine ß-hydroxylase (*DBH*) had the greatest number of sites altered by methionine. Examples of differentially methylated genes critical for early mammalian embryo development included negative elongation factor A (*NEFLA*) in the ICM, and Wnt family member 7A (*WNT7*) in the TE. 

### 2.6. Methionine Concentration Affects DNA Methylation of Specific Imprinted Genes

**Figure 6:**
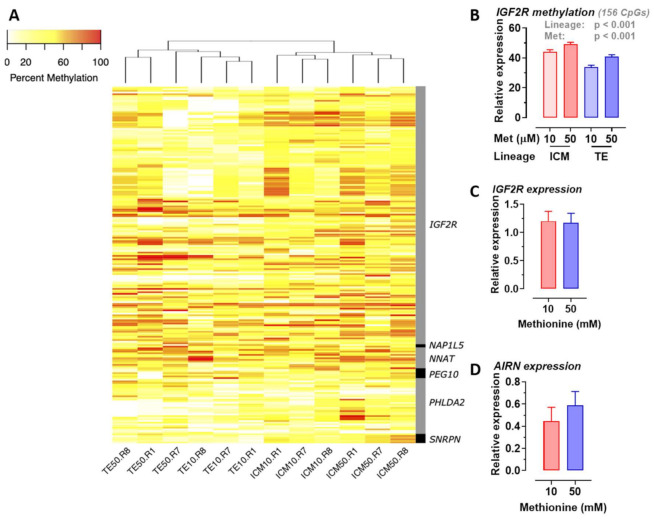
Effect of added methionine (50 v 10 µM) during in vitro oocyte maturation and embryo culture on (**A**) heat map of CpG methylation for six imprinted genes (paternally expressed 10, *PEG10*; nucleosome assembly protein 1 like 5, *NAP1L5*; insulin like growth factor 2 receptor, *IGF2R*; neuronatin, *NNAT*; small nuclear ribonucleoprotein polypeptide N, *SNRPN*; pleckstrin homology like domain family A member 2, *PHLDA2*) within the two primary cell lineages (inner-cell mass (ICM), and trophectoderm (TE)) of Day 8 bovine blastocysts; (**B**) CpG methylation of *IGF2R* at the two methionine concentrations within ICM and TE lineages; and transcript expression for *IGF2R* (**C**) and *AIRN* (**D**) in whole blastocysts.

Because genomic imprinting can be affected by embryo culture [35,36,37], we next determined the effects of methionine concentration during IVP on the methylation status of a subset 32 genes known to be imprinted in cattle (Table S5; http://igc.otago.ac.nz/). Of these, the methylation status of six genes (paternally expressed 10, *PEG10;* nucleosome assembly protein 1 like 5, *NAP1L5;* insulin like growth factor 2 receptor, *IGF2R;* neuronatin, *NNAT*; small nuclear ribonucleoprotein polypeptide N, *SNRPN;* pleckstrin homology like domain family a member 2, *PHLDA2*) was found differ (*p* < 0.001) between cell lineages (ICM v TE) and/or between added methionine concentrations (50 v 10 µM) (Figure 6A). Our attention was drawn to the 156 differentially methylated CpGs of insulin-like growth factor 2 receptor (*IGF2R*) (Figure 6B), the majority of which were hypomethylated at 10 v 50 µM at two clusters within the second intron differentially methylated region (DMR2) of this gene (Figure S5). The CpG island within DMR2 surrounds the promoter for a long non-coding RNA, *AIRN* (Figure S6), which is exclusively transcribed from the paternal allele within imprinted tissues and is thought to suppress *IGF2R* on the paternal allele by transcriptional interference [38,39]. However, in Day 8 blastocysts, transcript expression for *AIRN* and *IGF2R* did not differ between the two methionine concentrations (Figure 6C,D).

## 3. Discussion

A number of important inter-related findings emerge from this study, the most significant of which is that a physiologically relevant reduction in added methionine concentration (from 50 to 10 µM) in bovine embryo-culture media led to genome-wide alterations to DNA methylation in more than 1600 genes involved in cellular processes that included, perhaps unsurprisingly, protein catabolism and autophagy primarily within the inner-cell mass. The methylation status of a number of imprinted genes was also altered at the lower concentration of methionine. Our attention was drawn to the 156 differentially methylated CpGs within *IGF2R*, the majority of which were hypomethylated at two clusters within DMR2, which surrounds the promotor region of *AIRN*, a long non-coding antisense transcript the expression of which contributes towards the imprinted status of this gene. This is potentially significant because we previously demonstrated that loss of methylation at this locus following embryo culture is associated with a reduction in fetal *IGF2R* transcript and cation-independent mannose-6-phosphate receptor expression, which in turn is associated with an in utero-overgrowth syndrome, referred to as large offspring syndrome (LOS) [35]. These epigenetic modifications to DNA methylation in turn probably arose as a consequence of a reduced flux of methyl groups through the methionine cycle (Figure 1), represented by the allosteric up-regulation of *MAT2A* expression and MAT enzyme (EC 2.5.1.6) activity, increase in SAM:SAH ratio, and reduced intra-cellular concentration and egress of Hcy at the lower concentration (10 µM) of added methionine (Figure 5). In this respect it is possible that the bovine model, in which these experiments were conducted, was particularly sensitive to fluctuations in methionine concentrations within culture media as the absence of expression and activity of BHMT (EC 2.1.1.5), at least within the somatic-cell lineages of the ovarian follicle (Figure 1, Figure 2, Figure 3 and Figure 4), is expected to lead to greater fluctuations in SAM-mediate transmethylation reactions (Figure S1). However, the almost complete absence of BHMT expression in the somatic-cell lineages of the ovarian follicle for the other species tested (including human KGN cells but excluding rat granulosa cells) suggests that this enhanced sensitivity may be more generic among non-rodent species. This awaits confirmation, however, as does the full consequences of the epigenetic alterations to DNA methylation reported. Nevertheless, the current study supports the contention that mammalian gametes/embryos are epigenetically vulnerable to physiological fluctuations in 1C metabolic pathways.

### 3.1. Interspecific Variation in 1C Metabolism within the Ovarian Follicle and Preimplantation Embryo

The increased intracellular concentration of SAM, and increased SAM:SAH ratio, which followed 48 h culture of bovine granulosa cells in 10 µM added methionine (Figure 5) may, on first inspection, seem counter intuitive, but in fact reflects the adjustment made by these cells to chronic exposure to this low physiological concentration of methionine. Culture of human embryonic stem cells and induced pluripotent stem cells in the presence of reduced concentrations of methionine (from 120 µM to 12 µM) led to significant reductions in intracellular SAM and Hcy concentrations after 5 h; however, in contrast to Hcy (which remained low), SAM concentrations had returned to normal by 24 h [40]. Consistent with the current study, *MAT2A* expression was increased at the lower methionine concentration, and we can confirm that this leads to an increase in MAT enzyme activity (although we cannot dismiss a contribution from the *MAT1A* gene product). Transcripts for *MAT2A* were ubiquitously expressed in ovarian and embryonic cells in all species studied (Figure 2). In contrast, transcripts for *MAT1A* were restricted predominantly to hepatic cells, although low-level expression was detected in rat granulosa cells, all somatic-cell types in the bovine ovarian follicle and bovine oocyte. *MAT1A* transcripts and protein of maternal origin were previously reported in bovine embryos up to the 8-cell stage but not beyond [16]; an observation consistent with that of the current study. However, the importance of maternally inherited *MAT1A* and *MAT2A* isoforms in the lead up to embryonic genome activation is uncertain, as neither the inclusion of a *MAT2A* product inhibitor or methionine antagonist during the first three days of culture affected bovine embryo development to the morula stage [41,42]. These observations question the importance of methionine during the early cleavage stages of embryo development. Although the epigenetic consequences of methionine withdrawal during this period are not known, as an essential amino acid it is noteworthy that methionine is frequently, but not always, omitted in the first phase of commercially available sequential media used for extended culture to the blastocyst stage [25,43]. As methionine concentrations were altered throughout IVM, IVF, and IVC in the current study, we can provide no further insight into the temporal effects of its inclusion.

The current study extends that of Kwong et al. [14] by revealing a complete lack of BHMT protein expression and enzyme activity within the bovine ovarian follicle. In such circumstances, model [12] predictions (Figure S1) indicate a greater fluctuation in SAM-mediated transmethylation in response to methionine input, which inevitably becomes more reliant on vitamin B_12_ and folate-dependent methionine synthase (*MTR*) (EC 2.1.1.13) activity (Figure 1). The implications of this observation for bovine oocyte and subsequent embryo development, however, remains to be fully explored, as absence of *BHMT* transcript expression in bovine cumulus cells was matched by low-level *BHMT* expression in the oocyte (Figure 2). Furthermore, our failure to detect *BHMT* transcripts in bovine blastocysts (this study and [14]) is at odds with the report of Ikeda et al. [16] for bovine pre-hatching embryos; and functional MTR and BHMT mediated pathways have been shown to exist and contribute to SAM production in a partially redundant manner within the mouse blastocyst [18]. Interestingly, targeted BHMT knockdown and folate-cycle inhibition in that study indicated that, whilst both pathways are operative within the mouse ICM, only the folate pathway is operative within the mouse TE. Furthermore, it required disruption of both pathways to significantly reduce intracellular SAM levels and 5-methylcytosine immunofluorescence (5mC) within the ICM.

### 3.2. Methionine Concentration during In Vitro Embryo Production Affects Global DNA Methylation

In directly adjusting methionine concentrations within embryo culture media, the current study circumvented, to an extent, the BHMT and MTR mediated regulation of SAM investigated by Zhang et al. [18]; although altered flux of methyl groups through the methionine cycle (Figure 1) would inevitably have led to allosteric adjustments in the activity of these and other enzymes within this system (Figure S1). Using RRBS, we reveal that a reduction of added methionine (from 50 to 10 µM; equating to final media concentrations of 52–54 and 12–14 µM once methionine contributions from serum or albumin are considered (Figure S3)) led to a significant alteration in CpG methylation in >1600 genes across both ICM and TE lineages. Loss of methylation accounted for around 80% of these differences, with a 30% greater number of differentially methylated CpGs in the TE than in the ICM (Table 2).

Concentrations of methionine investigated in this phase of the study represent the physiological limits of what could be considered ‘normal’ in biological fluids [26], and are certainly well within the range of methionine concentrations encountered in commercially available cell and embryo culture media (Table S1). Importantly, the lower final concentration of methionine (i.e., ~14 µM) had only a very modest detrimental effect on embryo development (Table 1); an observation broadly in keeping with that of Bonilla et al. [19]. However, in contrast to the current study, Bonilla et al. [19] were unable to detect differences in DNA methylation by 5mC immunofluorescence; probably due to the lack of sensitivity of this approach compared to that used in the current study. Our observations on the effects of methionine restriction on embryo development are not without precedent. A subtle adjustment to the methionine level of dairy-cow diets (from 1.89 to 2.43% methionine) altered the expression of 276 genes in Day 7 blastocysts recovered from ovarian-stimulated dams [44], and restricted dietary methyl-donor (i.e., methionine and vitamin B_12_) provision up to Day 6 following insemination in sheep altered DNA methylation and led to hypertensive and insulin resistant offspring [4].

In the current study, pathways with the greatest number of differentially methylated genes of interest (GOI) within the ICM were broadly associated with proteolysis and autophagy (Tables S3 and S4). Notable genes included Calpain 2 and 7 (*CAPN2* and *CAPN7*), which are members of a ubiquitously expressed family of calcium-dependent cysteine proteases, mutations in which can be embryo lethal [45,46]. A separate cluster of genes included peptidase mitochondrial processing alpha subunit (*PMPCA*) and lon peptidase 1 mitochondrial (*LONP1*); these are involved in protein processing and degradation of misfolded proteins within the mitochondrion. Both are linked to mitochondrial function as well as endoplasmic reticulum stress in the mammalian embryo [47,48]. Autophagy related 4B cysteine peptidase (*ATG4B*) and ubiquitin specific peptidase 12 (*USP12*) are involved in removal of endogenous proteins and damaged organelles. They are intricately involved in cellular remodeling during differentiation [49]. Finally, *USP12,* together with BRCA1 associated protein 1 (*BAP1*) and lysine demethylase 8 (*KDM8*), is also involved in chromatin remodeling during embryogenesis [50,51].

Notable GO terms for the TE included ‘intracellular signal transduction’, ‘protein kinase activity’, and ‘pathways in cancer’ (Tables S3 and S4). Genes harboring the greatest number of differentially methylated CpGs within these terms included phospholipase C-like 2 (*PLCL2*), Wnt family member 7A (*WNT7A*), insulin-like growth factor 1 receptor (*IGF1R*), and mitogen activated protein kinase 1 (*MAPK1*). Collectively, these genes are involved in regulation of cell fate and proliferation during blastocyst formation [52,53,54,55].

Finally, KEGG ‘metabolic pathways’ harbored the greatest number of differentially methylated GOI common to both the ICM and TE (Tables S3 and S4). Notable genes from these lists are closely associated with 1C metabolism and include dopamine beta-hydroxylase (*DBH*; a member of the copper type II, ascorbate-dependent monooxygenase family), catechol-o-methyltransferase (*COMT*; catalyzes the transfer of a methyl group from SAM to catecholamines (e.g., dopamine)), lysophosphatidylcholine acyltransferase 1 (*LPCAT1*; involved in phospholipid metabolism, and is known to reduce lipotoxicity caused by oxidative stress in mouse oocytes [56]), and pipecolic acid and sarcosine oxidase (*PIPOX*; involved in sarcosine (N-methyl-glycine) metabolism). Interestingly, the expression both *MAT2A* and *COMT* were upregulated in human blastocysts derived from in vitro rather than in vivo matured oocytes [57].

### 3.3. Methionine Concentration during In Vitro Embryo Production Affects Methylation of Imprinted Genes

Reducing methionine concentrations during IVM, IVF, and IVC altered the methylation status of 6 out of 32 genes known to be imprinted in cattle (Figure 6; Table S5). The catalogue we interrogated was not exhaustive when it came to the list of imprinted genes for this species. It was not our purpose to establish the full extent of errors in genomic imprinting but merely, for illustrative purposes, to enquire if the methylation status of a reasonable cohort of imprinted genes could be altered. It is noteworthy, however, that four out of the six differentially methylated genes identified (i.e., *IGF2R*, *NAP1L5*, *NNAT*, and *SNRPN*) are epigenetically altered in LOS bovine fetuses [36], with the epigenetic status of the remaining two genes (*PHLDA2, PEG10*) linked to fetal and placental defects [58,59].

Our attention was drawn to the methylation status of DMR2 within *IGF2R* (Figure S5) because of its association with loss of imprinting in the LOS [35,36]. However, in Day 8 blastocysts, neither the expression of this gene nor the antisense transcript (*AIRN*), transcribed from DMR2 and participates in the monoallelic expression of *IGF2R* (at least in mice [60]), was altered (Figure 6). This observation was anticipated for the following reasons. Firstly, we and others had previously demonstrated that the monoallelic expression of *IGF2R* and several other imprinted genes in the sheep and cow occurs after the blastocyst stage at around the Day 21 (post implantation) [61,62]. Secondly, multiple sequence alignment revealed variable homology in DMR2 between species (Figure S6), suggesting that the precise mechanism of *IGF2R* imprinting may not be highly conserved. In support of this contention, Chen et al. [63] observed altered methylation at both DMR1 and DMR2 in LOS bovine fetuses and, intriguingly, no difference in *AIRN* expression. Regulation of the monoallelic expression of *IGF2R* and the effects of methionine deprivation during in vitro culture in this species, therefore, requires further investigation involving assessments made in the elongating trophoblast.

### 3.4. Concluding Remarks

The current study demonstrates that the mammalian oocyte and preimplantation embryo is acutely sensitive epigenetically to fluctuations in the provision of 1C substrates and/or cofactors, specifically methionine. It further indicates that the lack of BHMT activity, at least within the somatic compartment of the ovarian follicle, may increase this sensitivity. Further experimentation is required, however, to determine the spatial and temporal distribution of BHMT activity during oocyte maturation and preimplantation embryo development for all species studied, and to establish the extent of redundancy between the vitamin B_12_/folate-dependent MTR and choline/betaine-dependent BHMT pathways in driving SAM-mediated methylation of DNA and associated proteins.

## 4. Materials and Methods

Procedures described and reported in this article did not involve the use of live animals. All tissues were recovered post mortem. Approval was granted by the Animal Welfare and Ethical Review Board (AWERB) of the University of Nottingham (PDBF3E539; on 18/12/2018).

### 4.1. Tissue Collection and Primary Cell Isolation

All reagents were obtained from Sigma Aldrich (Poole, UK) unless otherwise stated. Tissues (liver and ovaries) from the three domestic species were recovered post mortem from a local abattoir and transported (in pre-warmed phosphate buffered saline (PBS) at 37 °C) to the laboratory ~1h away. Details of cell (oocyte, cumulus, granulosa, and theca) recovery were as described previously [64]. Briefly, granulosa cells were retrieved by scrapping and theca sheet layers peeled off from hemi-dissected follicles. Cumulus–oocyte complexes suspended in PBS/poly-vinyl alcohol (PVA) were denuded by vortexing for 3 min at room temperature. Purity of granulosa- and theca-cell populations was confirmed by qPCR for two steroidogenic enzymes (Figure S1). Rat granulosa cells (retrieved from ovaries *post mortem*) were aspirated using a 27G needle attached to a 1 mL syringe using a stereomicroscope with additional light arms. All cells were snap frozen and stored at −80 °C until analyses.

### 4.2. Cell Culture

Primary bovine granulosa-cells were cultured as described previously [65] with the following modifications. Cells were seeded at 3.5 × 10^5^ cells per well in fibronectin-coated (6µg/mL) 24-well plates containing 500 µl methionine-free DMEM/F-12 (Sigma, D9785) to which 10 or 50 µM methionine was added. Cells were cultured in a humidified atmosphere (38.5 °C, 5% CO_2_, 95% air) for up to 144 h, with cells and media harvested at 48, 96, and 144 h for 1C metabolite and transcript analyses, and at 48 h for MAT activity analyses.

HepG2 (human hepatocellular liver carcinoma cell line [28]) and KGN (human granulosa-like tumor cell line [27]) cells were obtained from American Type Culture Collection (HB-8065^TM^) and Rikken Cell Bank (Japan) respectively. Microsatellite genotyping for both cell types was performed by the European Collection of Authenticated Cell Cultures, Porton Down, UK to confirm purity. These cells were cultured in DMEM/F-12 containing 10% (*v/v*) fetal calf serum (FCS) in T75 flasks under humidified conditions (37 °C, 5% CO_2_, 95% air). Media were changed every 48 h and cell pellets washed in PBS prior to snap freezing for storage at −80 °C prior to transcript and protein analysis.

### 4.3. In Vitro Embryo Production (IVP), Immunostaining, and Sexing

Full details of IVP (embracing IVM, IVF, and IVC to the blastocyst stage) for bovine, ovine, and porcine embryos were described previously [14,29]. For bovine gametes/embryos cultured in 10 and 50 μM added methionine, the following modifications were made. Modified TCM199 41,150 methionine-free formulation was purchased from Gibco™ (Thermo Fisher Scientific, UK). Basal Eagle’s Medium (BME) [50X] essential amino acid supplement was custom-made to exclude methionine. Requisite concentrations of methionine were added to media and concentrations confirmed by HPLC analysis before use (Figure S3). In fact, the addition of 10% (*v/v*) FCS to IVM media contributed 3–4 µmol/L methionine, whereas the addition of 0.6% BSA (*w/v*) to IVF and 0.3% BSA (*w/v*) IVC media each contributed <2 µmol/L methionine, bringing actual final media concentrations to around 12–14 and 52–54 µM methionine. Grade 1 and 2 COCs [66] were matured in groups of 30 in 400 μL of modified TCM199 (10 vs. 50 μM added methionine) supplemented with 10% FCS for 22 h (38.8 °C, 5% CO_2_ in air). Matured COCs were inseminated with frozen-thawed bull sperm from a single sire at a final concentration of 1 × 10^6^ spermatozoa/mL TALP media supplemented with 10 or 50 μM methionine and fatty acid-free BSA.

Gametes were co-incubated in 50 μL drops under mineral oil for 20 h (38.8 °C, 5% CO_2_ in air). Presumptive zygotes were denuded and cultured in 400 μL synthetic oviductal fluid (SOF) media supplemented with custom-made BME (10 vs. 50 μM added methionine) and fatty acid-free BSA (0.3% *w/v*) until Day 8 post insemination (38.8 °C, 5% CO_2_, 5% O_2_, 90% N_2_). Media were renewed every 48 h. Cleavage was recorded on Day 2 post-insemination and blastocyst development recorded on Days 7 and 8 according to IETS criteria [67].

Day 8 blastocysts (*n* = 85) were immunostained according to Nichols et al. [68]. Primary antibody rabbit anti-human Nanoghomeobox (dilution 1:400, Santa Cruz, CA, USA) and secondary antibody Alexa Fluor™ 488 (dilution 1:500, catalogue number) was used to stain the epiblast. Primary antibody goat anti-human SOX17 (dilution 1:750, catalogue number) and secondary antibody Alexa Fluor™ 647 (dilution 1:500) was used to stain the hypoblast. Blastocysts were mounted on slides using FluoroShield™ with DAPI (Sigma Aldrich). Images were obtained using an epifluorescence microscope (Leica, DM4000 B; Wetzlar, Germany). Total, trophectoderm, inner-cell mass, epiblast and hypoblast cell counts were analyzed using FIJI software (https://imagej.net/ImageJ).

Day 8 bovine blastocysts (*n* = 71) underwent multiplex PCR using Y-chromosome specific and bovine specific primers (see Table S6 and Figure S7 for details). Specifically, sex determining region Y primers (*SRY*; NCBI GenBank EU581861.1) were designed using Primer Express software version 3.0.1 (Applied Biosystems, Warrington, UK). Bovine specific primers were selected according to the protocol described by Rattanasuk et al. [69]. Primers were supplied by Eurofins Genomics (Ebersberg, Germany).

### 4.4. Quantitative Real-Time PCR

Methodologies for RNA extraction, reverse-transcription, and quantitative real-time polymerase chain reaction (qPCR) adhered to protocols previously published by our group for ovarian-follicular cells, oocytes, and blastocysts [29,30]. For each of three replicates within each species, RNA was extracted from 20 mg liver, granulosa, and thecal cells from three antral follicles, cumulus cells from 150 cumulus–oocyte complexes, pools of 40 oocytes and 40 blastocysts, and 1 × 10^6^ HepG2 and KGN cells. For detection of 1C genes (*BHMT*, *MAT1A*, *MAT2A*), associated reference (*ACTB*) and steroid marker (*CYP17A1* and *CP19A1*) genes, transcript expression was determined using a Roche LightCycler 480 (Roche Diagnostics Ltd., West Sussex, UK) with gene specific primers and TaqMan probes (Table S2a).

Quantitative expression of the lineage specific marker gene in blastocysts (i.e., GATA Binding Protein 3; *GATA3*), transcripts of *IGF2R* and *AIRN*, together with corresponding reference genes (tyrosine 3-monooxygenase/tryptophan 5-monooxygenase activation protein zeta (*YWHAZ*), TATA-box binding protein (*TBP*), H2A.Z variant histone 1 (*H2AFZ*), and beta-2-microglobulin (*B2M*)) were performed using QuantiNova^®^ SYBR^®^ Green in the Bio-Rad thermal cycler CFX96 Real-Time System (Bio-Rad, Hercules, CA, USA). Primers were designed using Primer Express software version 3.0.1 (Applied Biosystems, Warrington, UK) and supplied by Eurofins Genomics (Ebersberg, Germany) (Table S2b). Data were normalized to the stable reference genes above using Reference Gene Selector tool in CFX Maestro™ Software based on GeNorm algorithm.

### 4.5. Western Blots (BHMT)

Protein extraction from liver, granulosa and theca cells involved homogenization (3 times at 1000 rpm for 20s) in lysis buffer (Tris-HCl, pH 6.8; Bio-Rad Laboratories Ltd., Hertfordshire, UK) containing 0.1% (*v/v*) protease inhibitor cocktail (Roche Diagnostics Ltd., West Sussex, UK) (Table S7A). Protein concentration was determined in the supernatant (Bradford assay, Bio-Rad Laboratories Ltd., Hertfordshire, UK). Proteins were denatured in 2X Laemmli buffer containing 1M reducing agent DTT at 100 °C for 5 min. A total volume of 20 µl of sample per well and 5 µl of molecular weight size marker was loaded on a 12% SDS-PAGE resolving gel with a 4% polyacrylamide stacking gel and proteins separated in 1X Tris/Glycine/SDS Running Buffer at 100 volt until samples reached the bottom of the gel. Transfer of proteins to membrane was confirmed by Ponceau red stain. Membranes were blocked in 5% (*w/v*) milk/0.1% (*v/v*) Tween-PBS (TPBS) overnight at 4 °C under rotation. This was followed by 2 h incubation with the primary antibody (in 5% (*w/v*) milk/TPBS) at room temperature under rotation. For human cell lines, incubation with primary antibody was performed overnight at 4 °C under rotation due to reported low abundance of protein of interest [70,71]. Antibodies and antibody concentrations are provided in Table S7.

### 4.6. Enzyme Activity Assays

BHMT activity: the assay, modified slightly from that of Lee et al. [17], is based on the detection of the products of transfer of ^3^H-methyl groups from ^3^H-betaine to Hcy, producing a mixture of ^3^H-dimethylglycine and ^3^H-methionine (Figure 1). The assay was validated for rat and bovine livers using different amounts of protein lysate (Figure 4a,b). The generation of labelled products increased linearly with increasing total liver protein in both species up to around 0.02 mg/mL protein.

MAT activity: This assay, based on that of Kotb and Kredich [72], relies on the conversion of ^35^S-methionine to ^35^S-Adeonsylmethionine (Figure 1). Briefly, activity was determined in 3.5–4.0 × 10^6^ bovine granulosa cells, which were homogenized in 50mM Tris-HCL containing 0.1% proteinase inhibitor. 0.185 MBq ^35^S-methionine was added to 0.1 mg/mL lysate and incubated at 37 °C for 20 min. ^35^S-Adeonsylmethionine was separated by HPLC and 3 mL Ultima Gold scintillation (Perkin Elmer, Coventry, UK) added to this fraction prior to counting.

### 4.7. LC-MS/MS

SAM, SAH, and Hcy metabolites were quantified based on the platform developed by Xu et al. [73] with the following modifications, which were specific for these metabolites. The LC-MS/MS system consisted of an ABI 4000 QTRAP tandem mass spectrometer employing an electrospray ion source (Turbo Ion Spray™) (SCIEX, Foster City, CA, USA) in positive ionization mode in combination with a LC-10AD LC system (Shimadzu, Kyoto, Japan) equipped with a SIL-HTC autosampler. The metabolites were separated on a SeQuant ZIC–pHILIC column (150 × 4.6 mm, 5 μm particle size) with guard column (SeQuant ZIC–pHILIC, 20 × 2.1 mm, 5 μm particle size). The column temperature was set at 45 °C and the flow rate was 0.4 mL/min. Good chromatographic separation was observed with a 13 min gradient program consisting of mobile phases solvent A (20 mM ammonium formate, pH3.5) and solvent B (100% acetonitrile). Elution commenced with 80% B for one min, a linear gradient from 80% B to 5% B for 5 min, followed by a linear gradient back to 80% lasting 2 min, then isocratic hold on 80% for another 5 min. MS parameters were set as follows: ion source temperature (450 °C), ion spray voltage (5000 V), curtain gas (25 psig), collision gas (8 psig), ion source gas 1 (20 psig), ion source gas 2 (20 psig) interface heater activated.

### 4.8. Reduced Representation Bisulfite Sequencing (RRBS)

Library preparation and sequencing: DNA extraction, restriction enzyme (MspI) digestion, end-repair, dA tailing, adaptor ligation, and bisulfite conversion were integrated into a single-tube reaction to minimize DNA loss based on the method of Guo et al. [74]. Unmethylated λ-DNA (8 pg/μL; Promega D1521) was spiked into samples before MspI digestion to monitor the extent of bisulfite conversion. Bisulfite converted DNA was purified using 10 ng protective carrier tRNA (Sigma Aldrich) before PCR amplification with KAPA HiFi uracil+ (Roche, Cape Town, South Africa) for 16 cycles. Amplified DNA fragments of 200–600 bp were size-selected and primer adapters removed using the BluePippin system. The final 12 quality-assured RRBS libraries, prepared from three replicates of ICM and TE (10 v 50 µM methionine) were multiplexed and sequenced using an Illumina NovaSeq™ 6000 (S1 flow cell) to achieve an average of ~78 million 150 bp paired-end reads (Edinburgh Genomics, Edinburgh, UK).

Bioinformatic analyses: multiplexed sequencing reads were trimmed to remove adapter sequences and low-quality bases using skewer with commands (-Q 20, -q 3) (https://source-forge.net/projects/skewer). Clean reads were aligned to the bovine reference genome (Bta.ARS-UCD1.2, April 2018) using bisulfite read mapper using default settings (Bowtie2) [75]. Duplicate reads were marked using MarkDuplicates (Picard tools,https://broadinstitute.github.io/picard/) and methylation values extracted using bismark_methylation_extractor module (commands --no_overlap --paired-end). The Bismark final alignment report for each experimental replicate can be found in Table S8. Methylation values were extracted from output SAM format files using methylKit (nolap = TRUE, mincov = 5, minqual = 20) [76]. To avoid identification of methylation differences that are related to underlying breed differences, bases at known variant positions were removed. Differentially methylated cytosines between groups were identified using the Chi-squared test in methylKit and annotated using the genomation package [76,77]. Results were filtered for ‘in gene/promoter’ to remove intergenic regions, and for a minimum difference of >20% methylation [78,79].

Gene set enrichment analysis (GSEA): based on the premise that regulatory regions of the genome that are important for gene transcription contain clusters of CpGs [80], genes that possessed ≥5 differentially methylated CpGs within a sliding window size of 1 kb were selected for GSEA (ICM, *n* = 179; TE, *n* = 267). Enrichment of gene ontologies (GO) and pathways using KEGG (Kyoto Encyclopedia of Genes and Genomes, https://www.genome.jp/kegg/) was performed using hypergeometric tests in the NIPA tool (https://github.com/ADAC-UoN/NIPA). Annotated pathways associated with ‘biological process’, ‘cellular component’ and ‘molecular function’ GO terms and pathways within the KEGG database were identified as significant based on functional enrichment of genes with differentially methylated clusters of CpGs. The Benjamini–Hochberg procedure [81] accounted for multiple testing, with a FDR ≤ 0.05 considered significant.

### 4.9. Statistical Analyses

Analyses were performed using the GenStat statistical package (19th Edition, VSN International, 2018; https://www.vsni.co.uk/). All proportion data, associated with embryo development, were analyzed using generalized linear models that assumed binomial errors and used logit-link functions. Terms fitted to these models were ‘replicate’ and ‘methionine concentration’. CpG methylation in individual imprinted genes was analyzed using restricted maximum likelihood (REML) mixed linear models. In these models, ‘individual CpG’ formed the random effect and ‘replicate’ + ‘methionine concentration x cell lineage’ were added as fixed effects. Data are presented as means ± SEM.

## Figures and Tables

**Table 1:** Effect of added methionine concentration on bovine in vitro embryo production.

Methionine (µM)	10	50	*p*-Value
Total oocytes matured	1269	1289	
**A. Embryo development**	19 replicates		
Cleaved of inseminated	0.803 ± 0.0108	0.779 ± 0.0111	-
Day 7 of: inseminated	0.115 ± 0.0073	0.129 ± 0.0075	-
:cleaved	0.143 ± 0.0089	0.165 ± 0.0095	-
Day 8 of: inseminated	0.214 ± 0.0086	0.240 ± 0.0098	0.053
:cleaved	0.267 ± 0.0104	0.307 ± 0.0109	0.015
Day 8 (IETS ^‡^ Stages 7–8) of			
:total blastocysts	0.570 ± 0.0167	0.627 ± 0.0157	0.017
**B. Day 8 blastomeres, *n***	3 replicates		
Total	95.1 ± 1.80	101.7 ± 1.53	0.006
Trophectoderm	69.0 ± 1.60	72.9 ± 1.32	0.065
Inner-cell mass	28.0 ± 1.01	30.0 ± 0.85	-
:Epiblast	12.7 ± 0.66	14.3 ± 0.59	0.080
:Hypoblast	15.0 ± 0.74	15.2 ± 0.59	-
**C. Sex distribution ^†^**	3 replicates		
Males of Day 8 blastocysts	0.577 ± 0.1159	0.611 ± 0.1157	-

^†^ Sex distribution established by PCR (Tables S6 and S7). ^‡^ IETS = International Embryo Transfer Society.

**Table 2:** Differentially methylated cytosine-phosphate-guanine dinucleotide (CpG) counts, transcripts and genes between added methionine concentrations (10 v 50 µM) within embryonic cell lineage (**A**), and embryonic cell lineage within methionine concentrations (**B**). Arrows indicate gain (↑) and loss (↓) of methylation at 10 compared to 50 µM methionine and in inner-cell mass (ICM) compared to trophectoderm (TE) cells (percentage altered in parentheses).

	A. Cell lineage		B. Methionine	
	ICM	TE	10 µM	50 µM
	10 v 50 µM	10 v 50 µM	ICM v TE	ICM v TE
**CpG count**	9991	13,123	12,213	8088
**↑ Methylation**	2449 (24.5)	2361 (18.0)	6671 (54.6)	3365 (41.6)
**↓ Methylation**	7542 (75.5)	10,762 (82.0)	5542 (45.4)	4723 (58.4)
**Transcripts**	1576	1743	1773	1427
**Genes**	1573	1738	1768	1425

## Data Availability

Raw sequence RRBS data (fastq format) is available at the European Nucleotide Archive (ENA) under project number PRJEB42616. Tables of differentially methylated CpG sites for each pairwise comparison are available at doi 10.6084/m9.figshare.13625870.

## Supplementary Materials

Supplementary Materials can be found at https://www.mdpi.com/1422-0067/22/4/1838/s1 [82,83].

## Abbreviations

1C	one carbon
5mC	5-methylcytosine
ACTB	actin βeta
BHMT	betaine-homocysteine methyltransferase
BME	basal medium eagle
COC	cumulus–oocyte complex
CpG	cytosine-phosphate-guanine dinucleotide
FCS	fetal calf serum
GOI	gene of interest
Hcy	homocysteine
HepG2	human liver cancer cell line
HPLC	high performance liquid chromatography
ICM	inner-cell mass
IVC	in vitro culture
IVF	in vitro fertilization
IVM	in vitro maturation
IVP	in vitro embryo production
KGN	human granulosa-like tumor cell line
LC-MS/MS	liquid chromatography-tandem mass spectrometry
LOS	large offspring syndrome
MAT1A	methionine adenosyltransferase 1A
MAT2A	methionine adenosyltransferase 2A
PBS	phosphate buffered saline
PVA	poly-vinyl alcohol
qPCR	quantitative real-time polymerase chain reaction
RRBS	reduced representation bisulfite sequencing
SAM	S-adenosylmethionine
SAH	S-adenosylhomocysteine
TE	trophectoderm
