# Supplementary material for: Interspecific Variation in One-Carbon Metabolism within the Ovarian Follicle, Oocyte, and Preimplantation Embryo: Consequences for Epigenetic Programming of DNA Methylation
Source: Int J Mol Sci. 2021 Feb 12;22(4):1838. doi: 10.3390/ijms22041838 (PMC7918761; doi:10.3390/ijms22041838)
Supplement: Supplementary file 1 [file ijms-22-01838-s001.pdf]

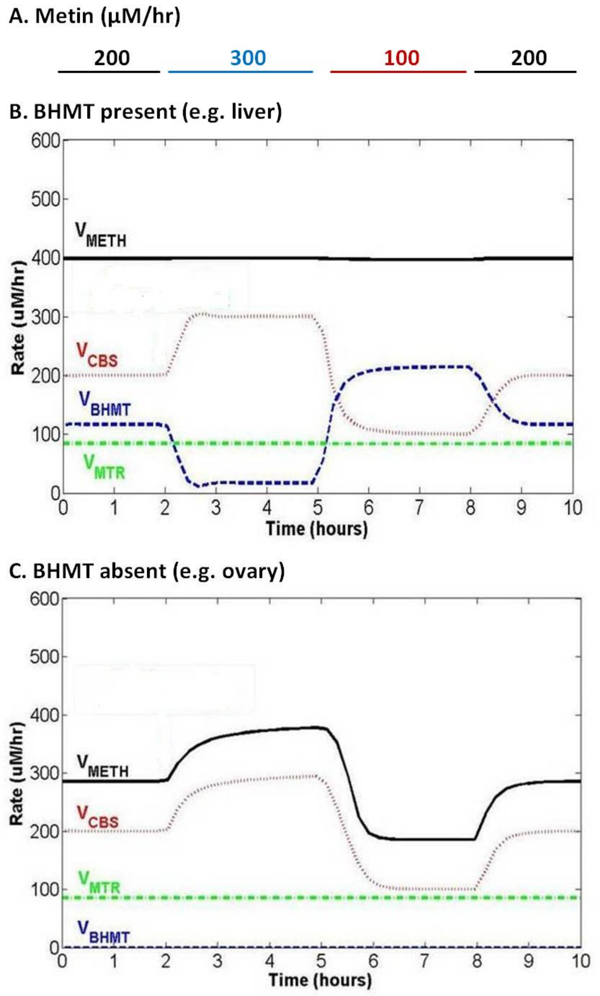

Figure S1. The effect of temporal variation in methionine input (Metin: μM/hr) (**A**) on rate of the sum of all S-Adenosylmethionine mediated methyltransferase reactions (V_{METH}), the reaction rates of cystathionine β-synthase (V_{CBS}), betaine-homocysteine methyltransferase (V_{BMHT}), and of methionine synthase (V_{MTR}) when BHMT is expressed and active, as in the liver (**B**), or when BHMT is not expressed/active, as in the bovine ovary (**C**). Simulations are based on the mathematical model described by Reed et al.[12], where 5-methyltetrahydofolate (the substrate for methionine synthase (MTR)) is held constant. In this model, BHMT was inactivated by setting V_{max} to zero (Equation 1). Simulations were generated using the software package Matlab and its inbuilt solver for stiff differential equations (ode15s):

https://www.mathworks.com/products/matlab.html.

$$\mathbf{V_{BHMT}} = \left(\boldsymbol{\delta}_1^{\mathbf{BHMT}} - \boldsymbol{\delta}_2^{\mathbf{BHMT}}\left([\mathbf{SAM}] + [\mathbf{SAH}] - \boldsymbol{\delta}_3^{\mathbf{BHMT}}\right)\right)\left(\frac{\mathbf{V_{max}^{BHMT}}[\mathbf{Hcy}]}{\mathbf{K_m^{BHMT}} + [\mathbf{Hcy}]}\right) \quad (\mathbf{Equation\ 1})$$

In Equation 1 V_{BHMT} = net rate of production by betaine-homocysteine methyltransferase; [Hcy] = homocysteine concentration; [SAM] S-adenosylmethionine concentration; [SAH] S-adenosylhomocysteine concentration; V_{max}^{BHMT} = maximum rate of enzyme reaction; K_m^{BHMT} = Michaelis constant; and δ_1^{BHMT}, δ_2^{BHMT}, δ_3^{BHMT} are constant parameter values used by Reed et al. (2004) [12].

The model predicts that, in cell types where BHMT is expressed, major changes in the system in response to fluctuating methionine input (Metin) are limited to the rates of transulfuration of homocysteine (V_{CBS}) and remethylation of homocysteine to methionine; the methylation rate (V_{MET}) remains virtually unaltered. In contrast, in cell types where BHMT is not expressed, the methylation rate (V_{MET}) is predicted to fluctuate with methionine input (Metin).

Table S1. Methionine concentration in commercially available cell, oocyte maturation and embryo culture media. Adapted from source(s): Sigma Aldrich (https://www.sigmaaldrich.com) [24, 25].

Cell culture	Methionine (µM)
Lebovitz L-15	502.6
Waymouth's 752/1 MB	335.1
DMEM	201.1
IMDM	201.1
TCM199	100.5
MEM	100.5
RPMI-1460	100.5
Williams' E	100.5
McCoy's 5A	100.0
BME	50.3
MDCB	30.0
Ham's F-10	30.0
Ham's F-12	30.0
NCTC-109	29.8

Embryo culture	Methionine (µM)
InVitroCare IVC1	0.0
InVitroCare IVC3	100.0
Origio ISM1	89.0
Origio BA	54.0
Vitrolife G-1™	0.0
Vitrolife G-2™	63.0
Sage QACM	0.0
Sage QABM	56.0
Cook SICM	4.0
Cook SIBM	43.0
Irvine CSC	53.0
IVFOnline Global	51.0

Abbreviation(s): BA, BlastAssist; BME, Basal Medium Eagle; CSC, Continuous Single Culture; DMEM, Dulbecco's Modified Eagle's Medium; IMDM, Iscove's Modified Dulbecco's Medium; ISM1, Innovative Sequential Media 1; MEM, Minimum Essential Medium; QABM, Quinn's Advantage Blastocyst Medium; QACM, Quinn's Advantage Cleavage Medium; RPMI, Roswell Park Memorial Institute Medium; SIBM, Sydney IVF Blastocyst Media; SICM, Sydney IVF Cleavage Media; TCM199, Tissue Culture Medium 199

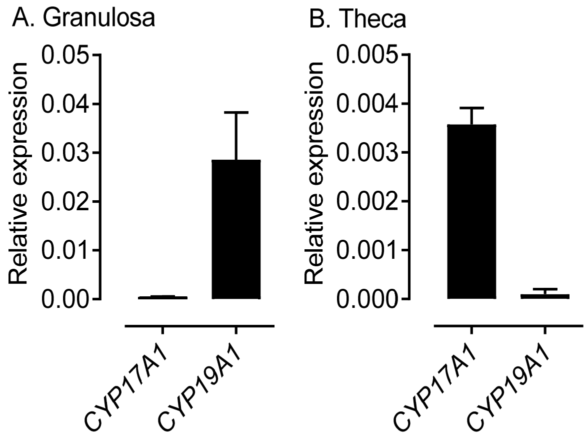

Figure S2. Relative (to *ACTB*) transcript expression (means ± SEM) for two steroidogenic enzymes (17α-hydroxylase and aromatase) to confirm purity of bovine theca- and granulosa-cell populations respectively for use in 1C transcript analyses. Similar confirmatory quality checks were performed for ovine and porcine theca and granulosa cells (data not shown). See Table S2a for details of qPCR.

Table S2a. List of primers and Taqman probes and Accession numbers used for detection of 1C genes in somatic cells, oocytes and embryos from the four species studied.

Gene	Primers and probe (5'-3')	NCBI accession no.
**BOVINE AND OVINE**		
BHMT		
FP	AGAGAAAATATCCGGGCAGAAAG	NM_001011679
RP	TCACACCCCCTGCTACCAAA	Bos Taurus
Probe	**FAM**-AATGAAGCCGCTTGTGACATTGCCC-**TAMRA**	
MAT1A		
FP	ACGGTGCGGTCATCCCTAT	NM_001046497
RP	CCAGCGTTATGTCTTCGTTGTG	Bos Taurus
Probe	**FAM**-CCATACCGTCGTCATCTCCGTGCA-**TAMRA**	
MAT2A		
FP	AGTGCCCAAAAAGCTTAAATATTGA	NM_001101131
RP	CTTTCCCGCAGAGCTTGAGG	Bos Taurus
Probe	**FAM**-TGTTAGCCTTTTTTCCCCAGACTTGTTGG-**TAMRA**	
ACTB		
FP	TGTGCGTGACATCAAGGAGAA	AF129289
RP	CGCAGTGGCCATCTCCTG	Ovis aries
Probe	**FAM**-CTGCTACGTGGCCCTGGACTTCGA-**TAMRA**	
CYP17A1		
FP	TCATCTCGCCATCGCCATCGTTAAGCT	NM_001009483
RP	CGGGCTAGCATCTCACCTACA	Ovis aries
Probe	**FAM**-TTGCCCTTTGGAGCCGGACCC-**TAMRA**	
CYP19A1		
FP	TGGGTTGCCATTGCCTTC	NM_001123000
RP	GGACAGTAAGGAGCTGGAGTGAG	Ovis aries
Probe	**FAM**-CCGTTGGAAAAGACAAGTCACCAGCAA-**TAMRA**	
**PORCINE**		
BHMT		
FP	CCTCAGAGCCGGATCGAAT	U53421
RP	CCCCTGTTCTCCAGCTTGTC	Sus scrofa
Probe	**FAM**-TCATGCAGACCTTCACCTTCTATGCCAGT-**TAMRA**	
MAT1A		
FP	TGCAGTACACACAGGACAATGG	XM_001925608
RP	GCACGGAGATGACGATGGT	Sus scrofa
Probe	**FAM**-CCGTCATCCCCGTGCGCA-**TAMRA**	
MAT2A		

FP	AGTGCCCAAAAAGCTTAAATATTGA*	NM_001101131
RP	CTTTCCCGCAGAGCTTGAGG*	Bos Taurus
Probe	**FAM**-TGTTAGCCTTTTTTCCCCAGACTTGTTGG-**TAMRA***	
ACTB		
FP	TGTGCGTGACATCAAGGAGAA	AF129289
RP	CGCAGTGGCCATCTCCTG	Ovis aries
Probe	**FAM**-CTGCTACGTGGCCCTGGACTTCGA-**TAMRA**	
CYP17A1		
FP	TGGCCCAGACCACAATTTAAA	NM_214428
RP	CCCAAAGATGTCCGCAACA	Sus scrofa
Probe	**FAM**-CTGCTTTCAGACAGACACATGCTCGCC-**TAMRA**	
CYP19A1		
FP	TCGTGCATAAAGTCCAGGGTTA	NM_214431
RP	CTGTACAGCCAAGAAATCTTAAAGAAGA	Sus scrofa
Probe	**FAM**-CATGGCAAGCTCTCCTTCTCAAACCAGA-**TAMRA**	
**HUMAN**		
BHMT		
FP	TGTGGAGCACCCAGAAGCA	NM_001713
RP	GAAGGTCTGCATGACGTTTGAG	Homo sapiens
Probe	**FAM**- CGCCAGCTTCATCGAGAGTTCCTCAG-**TAMRA**	
MAT1A		
FP	GGCTATGCTACCGACGAGACA	NM_000429
RP	CGGGCGTTGAGCTTGTG	Homo sapiens
Probe	**FAM**- AGTGCATGCCCCTCACCATCATCCT-**TAMRA**	
MAT2A		
FP	TTTAACCATTGTCTTGGCACACA	NM_005911
RP	TCGATCCTGCATATACTGCACAGT	Homo sapiens
Probe	**FAM**- ATGGCACTTTGCCTTGGTTACGCCC-**TAMRA**	
ACTB		
FP	CCTGGCACCCAGCACAAT	NM_001101
RP	GCCGATCCACACGGAGTACT	Homo sapiens
Probe	**FAM**- ATCAAGATCATTGCTCCTCCTGAGCGC-**TAMRA**	
**RAT**		
BHMT		
FP	TGCCACCAGATGGGATATTCA	NM_030850 Rattus
RP	GCAGCCGCCAATGTACCT	norvegicus
Probe	**FAM**- AAATACGCCAGAGAGGCCTACAACCTG-**TAMRA**	
MAT1A		
FP	GTTCAGTACGTGCAGGATAATGGT	NM_012860 Rattus
RP	TTCGTTGTGTTGCACAGAGATG	norvegicus
Probe	**FAM**- CTGTCATCCCTGTTCGCGTCCACAC-**TAMRA**	
MAT2A		
FP	CATCCAGATAAGATTTGTGACCAAA	NM_134351
RP	AGCAACAGTTTCACAAGCCACTT	Rattus norvegicus
Probe	**FAM**-CCTTGATGCACACCTTCAGCAGGACC -**TAMRA**	
ACTB		
FP	TCTGTGTGGATTGGTGGCTCTA	NM_031144 Rattus
RP	CTGCTTGCTGATCCACATCTG	norvegicus
Probe	**FAM**- CCTGGCCTCACTGTCCACCTTCCA-**TAMRA**	

Table S2b. List of SYBR Green primers and accession numbers used for detection of the lineage-specific marker in bovine blastocysts (*GATA3*) and associated reference genes, together with *IGF2R* and *AIRN* transcripts.

Gene		Primer sequence (5'-3')	Product (bp)	NCBI accession no.
GATA3	FP	AACATCGACGGTCAAGGCAA	217	NM_001076804.1
	RP	GGTGGATGGACGTCTTGGAG		
YWHAZ	FP	GATATCTGCAATGATGTACTGTCTCTTTT	107	NM_174814.2
	RP	CGGTAGTAGTCTCCTTTCATTTTCAA		
TBP	FP	GAATATAATCCCAAGCGTTTTGCT	103	NM_001075742.1
	RP	TGGCTCCTGTGCACACCAT		
H2AFZ	FP	GCAGGAAATGCATCGAAAGAC	126	NM_174809.2
	RP	AATGACACCACCACCAGCAATT		
B2M	FP	ATCCAGCGTCCTCCAAAGATTC	132	NM_173893
	RP	CTCCCCATTCTTCAGCAAATCG		
IGF2R	FP	GCAGCCTGTATACCCATCCC	152	NM_174352.2
	RP	ATCAAACACGTACCCGCTGT		
AIRN	FP	GTGATCAACCTGGATTGCTGC	185	NR_104052.1
	RP	AAGCCTGGGATTCTGACTGG		

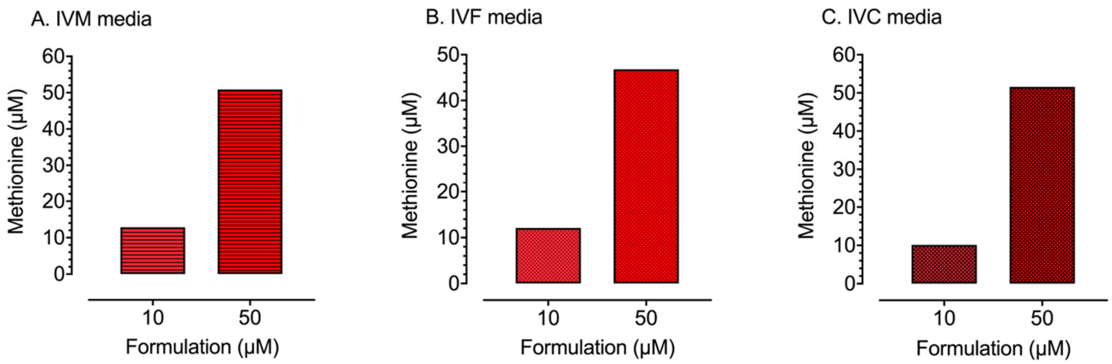

Figure S3. Embryo production media concentrations of methionine confirmed by HPLC analysis. *In vitro* maturation media (A); *in vitro* fertilisation media (B); *in vitro* culture media (C).

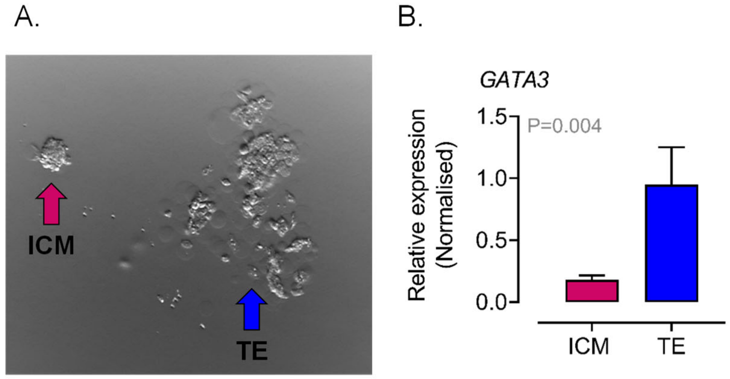

Figure S4. Immunodissection of primary cell lineages in Day 8 bovine blastocysts by the method of Tutt et al. [82]. Transmission microscopy shows isolation of inner-cell mass (ICM) and trophectoderm (TE) (A). Relative expression of TE marker, *GATA3*, in separately pooled ICM and TE cell samples following dissection (B). Validation conducted over five replicates and data presented as mean ± SEM.

Table S3. Top 5 enriched Gene Ontology (GO) terms and KEGG pathways ranked by number of differentially methylated genes of interest (GOI). Abbreviation(s): GOI, genes of interest; FDR, false discovery rate; ICM, inner cell mass; TE, trophectoderm. Venn diagram: pathways enriched in ICM (red circle); pathways enriched in TE (blue circle); pathways enriched in ICM and TE (purple intersection).

A. Biological Process GO

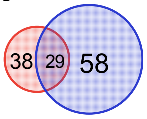

Inner-cell mass ICM (*n*=38)	GOI	FDR (q-value)
GO:0006508 Proteolysis	11	0.0456
GO:0050821 Protein stabilisation	5	0.0060
GO:0006914 Autophagy	4	0.0060
GO:0010468 Gene expression	4	0.0433
GO:0046854 Phosphatidylinositol phosphorylation	3	0.0060

Trophectoderm TE (*n*=58)	GOI	FDR (q-value)
GO:0035556 Intracellular signal transduction	11	0.0326
GO:0010628 Regulation of gene expression	9	0.0207
GO:0018108 Peptidyl-tyrosine phosphorylation	5	0.0148
GO:0048490 Anterograde synaptic transport	3	0.0013
GO:0008089 Anterograde axonal transport	3	0.0024

ICM and TE (*n*=29)	GOI		FDR (q-value)	
	ICM	TE	ICM	TE
GO:0016310 Phosphorylation	10	13	0.0168	0.0256
GO:0048015 Phosphatidylinositol signalling	4	3	0.0006	0.0096
GO:0043410 Regulation of MAPK cascade	3	4	0.0154	0.0149
GO:0045766 Regulation of angiogenesis	3	4	0.0221	0.0233
GO:0019722 Calcium signalling	2	5	0.0367	0.0025

B. Cellular Component GO

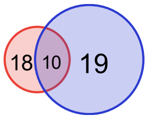

Inner-cell mass ICM (*n*=18)	GOI	FDR (q-value)
GO:0005829 Cytosol	40	0.0009
GO:0005815 Microtubule organisation	6	0.0069
GO:0030424 Axon	5	0.0359
GO:0062023 Collagen containing ECM	3	0.0179
GO:0005930 Axoneme	3	0.0150

Trophectoderm TE (*n*=19)	GOI	FDR (q-value)
GO:0005856 Cytoskeleton	13	0.0393
GO:0005623 Cell	8	0.0397
GO:0030496 Midbody	6	0.0127
GO:0098978 Glutamatergic synapse	6	0.0391
GO:0031514 Motile cilium	4	0.0165

ICM and TE (*n*=10)	GOI		FDR (q-value)	
	ICM	TE	ICM	TE
GO:0005770 Late endosome	4	4	0.0089	0.0338
GO:0030667 Secretory granule	3	4	0.0003	0.0001
GO:0070062 Extracellular endosome	3	3	0.0075	0.0268
GO:0030659 Cytoplasmic vesicle	3	3	0.0138	0.0435
GO:1904115 Axon cytoplasm	2	4	0.0149	0.0040

C. Molecular Function GO

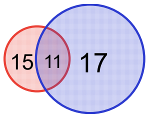

Inner-cell mass ICM (*n*=15)	GOI	FDR (q-value)
GO:0016787 Hydrolase activity	18	0.0417
GO:0008233 Peptidase activity	9	0.0186
GO:0008234 Cysteine-type peptidase activity	5	0.0033
GO:0004175 Endopeptidase activity	4	0.0052
GO:0008201 Heparin binding	4	0.0110

Trophectoderm TE (*n*=17)	GOI	FDR (q-value)
GO:0004672 Protein kinase activity	15	0.0392
GO:0004713 Protein tyrosine kinase activity	4	0.0447
GO:0004402 Histone acetyltransferase activity	3	0.0106
GO:0004714 Receptor protein tyrosine kinase	3	0.0279
GO:0022849 Glutamate-gated ion channel activity	2	0.0001

ICM and TE (*n*=11)	GOI		FDR (q-value)	
	ICM	TE	ICM	TE
GO:0016301 Kinase activity	9	12	0.0291	0.0412
GO:0030165 PDZ domain binding	2	3	0.0225	0.0171
GO:0008081 Phosphoric diester hydrolase	2	3	0.0432	0.0351
GO:0015485 Cholesterol binding	3	2	0.0033	0.0478
GO:0005078 MAP-kinase scaffold	2	2	0.0002	0.0007

D. KEGG Pathways

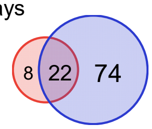

Inner-cell mass ICM (*n*=15)	GOI	FDR (q-value)
bta00562 Inositol phosphate metabolism	3	0.0349
bta04152 AMPK signalling pathway	3	0.0457
bta04136 Autophagy – other	2	0.0349
bta00513 N-Glycan biosynthesis	2	0.0361
bta03050 Proteosome	2	0.0361

Trophectoderm TE (*n*=17)	GOI	FDR (q-value)
bta05200 Pathways in cancer	13	0.0072
bta04151 PI3K-Akt signalling	10	0.0078
bta04010 MAPK signalling	9	0.0065
bta04014 Ras signalling	8	0.0065
bta05166 HTLV-I infection	8	0.0065

ICM and TE (*n*=22)	GOI		FDR (q-value)	
	ICM	TE	ICM	TE
bta01100 Metabolic pathways	17	25	0.0457	0.0157
bta05205 Proteoglycans in cancer	6	10	0.0348	0.0010
bta04360 Axon guidance	4	6	0.0449	0.0112
bta04140 Autophagy – animal	4	5	0.0351	0.0133
bta04390 Hippo signalling pathway	4	5	0.0361	0.0161

Table S4. Genes of interest (GOI) with the highest number of differentially methylated sites (DMS) selected from each Gene Ontology (GO) term and KEGG Pathway.

Pathways enriched in ICM		Differentially methylated GOI		CpGs	Promoter	Exon	Intron	CGI	Shore
BP	GO:0006508 Proteolysis	*PMPCA*	Peptidase, mitochondrial processing alpha subunit	48	1	22	27	24	20
CC	GO:0005829 Cytosol	*NELFA*	Negative elongation factor complex member A	50	1	15	35	31	10
MF	GO:0016787 Hydrolase	*PLCL2*	Phospholipase C like 2	92	0	9	85	38	19
KEGG	bta00562 Inositol phosphate metabolism	*PLCG1*	Phospholipase C gamma 1	24	0	6	18	10	5

Pathways enriched in TE		Differentially methylated GOI		CpGs	Promoter	Exon	Intron	CGI	Shore
BP	GO:0035556 Signal transduction	*PLCL2*	Phospholipase C like 2	118	0	6	112	51	16
CC	GO:0005856 Cytoskeleton	*FARP1*	FERM, ARH/RhoGEF and pleckstrin domain protein 1	49	1	3	46	5	17
MF	GO:0004672 Protein kinase	*IGF1R*	Insulin like growth factor 1 receptor	61	1	19	57	29	11
KEGG	bta05200 Pathways in cancer	*WNT7A*	Wnt family member 7A	69	0	3	57	16	31

Pathways enriched in ICM and TE		Differentially methylated GOI		CpGs		Promoter		Exon		Intron		CGI		Shore	
				**ICM**	**TE**	**ICM**	**TE**	**ICM**	**TE**	**ICM**	**TE**	**ICM**	**TE**	**ICM**	**TE**
BP	GO:0016310 Phosphorylation	*TOLLIP*	Toll interacting protein	70	83	4	4	32	31	38	47	23	39	34	34
CC	GO:0005770 Late endosome	*IGF2R*	Insulin like growth factor 2 receptor	25	51	0	11	7	5	18	46	8	32	10	12
MF	GO:0016301 Kinase activity	*PRKAR1B*	Protein kinase cAMP-dependent type 1 subunit β	56	81	0	1	6	15	53	75	35	45	16	32
KEGG	bta01100 Metabolic pathways	*DBH*	Dopamine β-hydroxylase	131	146	5	9	22	24	103	115	38	55	50	61

Abbreviation(s): BP, Biological Process; CC, Cellular Component; CGI, CpG island; ICM, inner cell mass; KEGG, Kyoto Encyclopedia of Genes and Genomes; MF, Molecular Function; TE, trophectoderm. † DMS distribution across genomic regions show an excess of annotation due to overlapping genes and inaccurate annotation of promoter regions in Bos taurus genome (Bta.ARS-UCD1.2, April 2018).

Table S5. Known imprinted genes in cattle. Adapted from the Catalogue of Imprinted Genes (http://igc.otago.ac.nz/home.html).

Imprinted genes								
Chr	Gene		Chr	Gene		Chr	Gene	
**4**	***PEG10***	♀	18	*PEG3/USP29*	♂	21	*RTL1*	♀
4	*PON3*	♂	18	*ITUP1*	♀	21	*PERK10/MEG8*	♂
4	*MEST*	♀	18	*PEG3*	♀	29	*CDKN1C*	?
**6**	***NAP1L5***	♀	18	*ZIM2*	♂	29	*H19*	♂
**9**	***IGF2R***	♂	21	*GTL2/MEG3*	♂	29	*IGF2*	♀
9	*PLAGL1*	♀	**21**	***SNRPN***	♀	**29**	***PHLDA2***	♂
13	*GNAS*	♂	21	*MEG8*	♂	X	*XIST*	♀
**13**	***NNAT***	♀	21	*MIRG/MEG9*	♂	?	*MAGEL2*	♀
14	*DGAT1*	?	21	*MKRN3*	♂	?	*COPG2IT1*	♂
18	*MIMT1*	♂	21	*UBE3A*	♂	?	*TSSC4*	♂
18	*USP29*	♂	21	*XLOC*	♀			

Imprinted genes with differentially methylated CpGs in the current study are coloured in **blue**. Imprinted genes associated with disorders in humans (e.g. Beckwith-Wiedemann, Prader-Willi/Angelman and Silver-Russell syndromes) and large animals (Large Offspring Syndrome) are shaded in grey [59, 83]. Paternally imprinted gene (♂), maternally imprinted gene (♀).

A

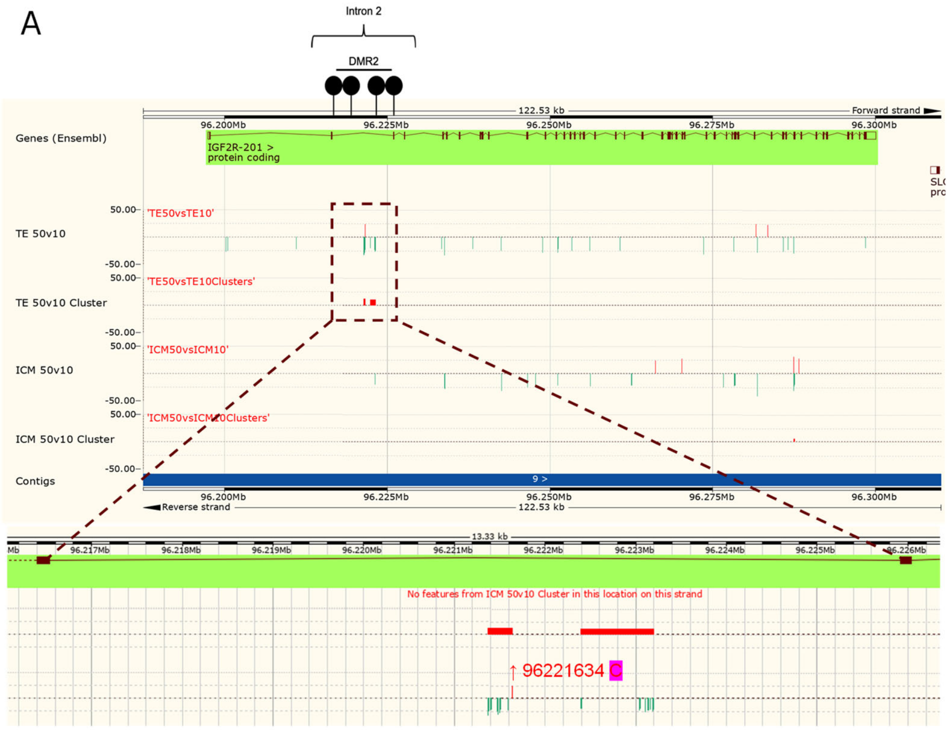

B

```
AAGGTGCTGAGAAGGCTCGGCGCCCGCAGGCTGGGCCCCGTGGCCGCCGGCGGAGGCGCG
GTCGCCAGGCCGAGCAGCCTCAGCGAGGTCGGGTTGCGAGCTCGGCCGGGCTCGGCCGCG
AGCGCCGAGGGCGGCAGGCGAGGCCCGGCCGGCCTGGCACGCGGCCTGGTCGGGCGGACT
CTGGTGAGCGCGGCCGAGCGCGCAGGGTCTGCAGGACCCGGCGTGGCCTGGCCGGCGGCG
CGTGGCTGGGGCTGGCGGGCGCGGGCGAGCGCTGCCGGACGGGCGCCCTGGCGCGCAGGG
TCGAGAGGACCCGGCGCGGCCCGGGCTGCAGAGCGTGGCTGGGTCTGACGGGCTCGGGCG
AGCGTGGCCTGGCGGGAGCCCTGGCGTGCAGGGTCGGGAGGACCTCGCGGGGCCTGGCCG
GTAGCGTGTGGCCGGGTCTGGTGGGTCCGGGTGAACGTGGCCTGGTCTGGCGGGCCCGGG
CGAGCGCGGCCTGGAGAGCCCGGCCTGGAGAGCGCTGTCTGGAGGGCCCAGCGCGTGGTC
TGGCGGACCCGGCGCGGTCTGGCGGCCCCGGCCTGGAGACCGCGATCGGGTGGACCCGCC
ACTGCCTGGCGGGCTCGGCAAGTGCGGTTTGGTCTGGCGGGCCCAGGCGAGCGCGGCTCG
GTCTGGGGGGCTCTGCCTGGAGGCTGCGGTCGGACAGGTCTGGCAGGCCCTGGACGCGCG
GCCTGGTCTGGAGGACCCGGCCTGGAGAGCGCGGCCTGGAGGACCCGGCGCGGTCTAGTC
TGGTGGGGTCTCGCCAGTGCTGTCTGGTCTGGTGGGCCCGGGTGAGCTCGGCCTGGTCTG
GCGGACCTGGCCTGGAGAGCGCCGTCTGGAGGACCCGGCGCGGCCTGGCGGGCCCTGGCG
AGCGCGGCCTGGCGGGCCCGGGGCGCAGGGTCTGAAGGACCCGGCGTGGCCTGGCTTGGA
GAGCTCGGCCTGGTCTGGCGGACCCGGCCTGGAGAGCGCCGTCTGGTGGGCCTGGCGCGC
GTGGCCTCGTTTGGAGGACCCGGCCTGGAGACCGTGATCTGGAGGGCCTGGAGCGCACGG
TCTGGCGCGGTCCGGAGGACCCGGCGCGATCTGGTGGGCCCGGTGAGCGCGAGCTGGTCT
GGTGGGCCCAGCGCGCGCGGCCTGGTCTGGTGGACCCGGCCTGGAGAGCGTGGTCTGGAG
GACCCGGCGCGCGCGGTCTGGCGGGCCTGGCGCGGTCCGGTTGGCCGAGCGCGCGGTCTG
GAGGACCCAGCGCGGTCTGGCGGACCCGGCCTGGAGAGCGCGGTCTGGAGGACCCGGCGC
GGCCTGGCGGGCCTGGGCGGACGCGGCCTGGTCTGGCGGGCCCTGCCGGGAGACCGCGGT
CTGGCCGGCACGGCGCGCGCAGCCCGGTCTGGAGGACCCGGCCTGGAGAGCGCGGCCTGG
AGGACCCGGCGCGGTCTGGTCTGCTGGGGCCCCGCCAGTGTGGTCTGGTCTGGCGCGCCC
GGCCTGGAGAGCGTGGTCTGGCGGGCCCGGCGCGCGGTCTGGAGGACCCGGCGCGATCTG
GCGGGCCCGGCGAGCGCGGCCCAGCCCATTTGGCGCCGTCCTCGGGAGCTGGCCGTGGGC
CTGGCGTGCTGGCCGCCGCCCCGCAGTCCTCGCCCGGCCTTCGTCCGGGGAGGACGGCCA
GGCTGGCCGGGGTGCCGCGGGAGTCCGCCGCGGGGGCGCTGCCGCGGTGCACCGCGGCCT
CTTGCCGAGTGCCGGGGTGGGCAATCGGTCCTGGCCAGTAGGCGGAGGAGCGGGCTGCAG
GCAGGCTGGACGGGCGAGCGGGCGCGGTGCCGATGGGGCCTGACGCTGAGGGACGCCGCT
GTCCCCAGCGCCGCGGCCGCGGAGCTCGGCCGGTCCGTCTCGGCTCAAGGCCAGGGCCCG
```

Figure S5. Two clusters of CpGs were hypomethylated within DMR2 of the *IGF2R* gene in the trophectoderm (TE) lineage following bovine embryo culture in low physiological methionine (10 v 50 μM). Bedgraphs demonstrate loss (↓, hypomethylated) and gain (↑, hypermethylated) of methylation at individual cytosine residues, and location of CpG clusters (**A**). Nucleotide sequence showing the two clusters in red. Hypomethylated cytosines in blue and hypermethylated cytosine (position 96221634) in pink C (**B**).

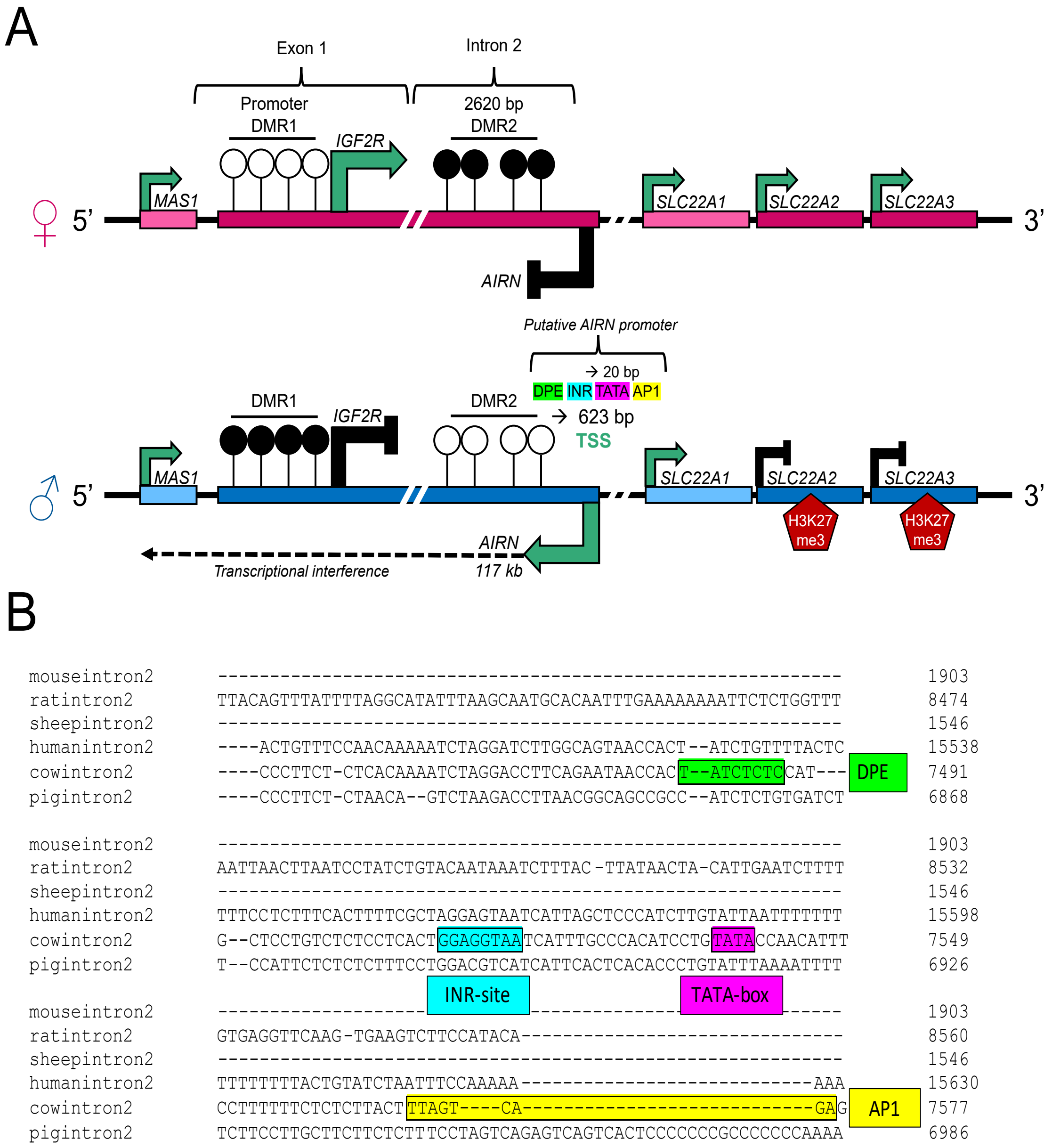

Figure S6. Bovine *IGF2R* gene is imprinted by antisense transcript, *AIRN*. Bovine genomic region on Chromosome 9 specific to *IGF2R* and *AIRN* (**A**). Multiple sequence alignment for *Igf2r*/*IGF2R* intron 2 with putative transcription initiation site and consensus binding site of core promoter elements in the bovine genomic region. Nucleotide numbers refer to location on chromosomes (**B**). Abbreviation(s): AP1, activator protein 1; DPE, downstream promoter element; INR, initiation response element.

Embryo sex determination

After ZP removal, individual blastocysts were washed in PVP/PBS (0.1% w/v), transferred in 2 µl to PCR tubes, and stored at -20°C until PCR.

Amplification reactions were conducted in a total volume of 25 µl containing 12.5 µl ImmoMix™ Red (Bioline), 0.125 µl BSP primers, 1.25 µl SRY primers, 7.75 µl of RNase-free water and 2 µl DNA template. Positive control DNA was extracted from male bovine liver and female bovine granulosa cells. Amplification was carried out in an Eppendorf AG 22331 thermocycler (Hamburg, Germany) with an initial denaturation at 95°C for 10 min followed by 38 cycles: 95°C for 30s, 55°C for 30s, 72°C for 1 min, with a final extension at 72°C for 7 min. 10 µl of PCR products were electrophoresed on 1.6% agarose gel and stained with ethidium bromide. DNA bands were visualised under an ultraviolet illuminator.

Table S6. Primers for sex determination of bovine blastocysts

Gene		Primer sequence (5'→3')	Product (bp)	NCBI accession no.
SRY	FP	TGAAACAAGACCAAAACCGGG	339	EU581861.1
	RP	TCCATGGACTTGCTCTACTGT		
BSP	FP	TTTACCTTAGAACAAACCGAGGCA	538	Rattanasuk *et al.* [69]
	RP	TACGGAAAGGAAAGATGACCTGAC		

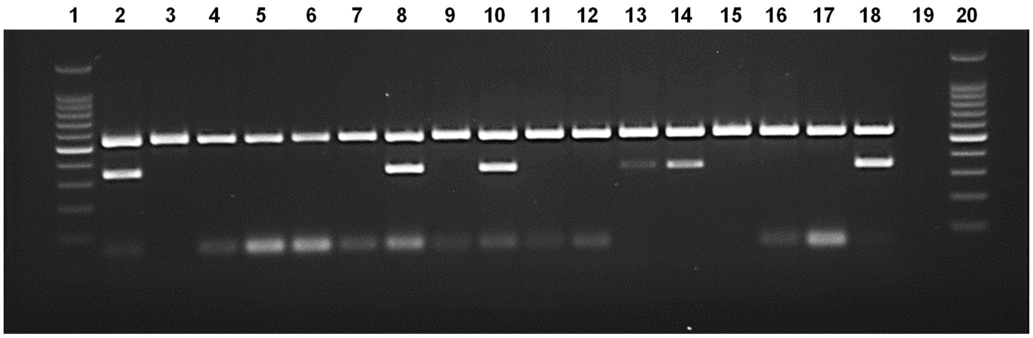

Figure S7. Bovine embryo sex distribution using the SRY/BSP-based method. Lane 1 and 20: 100 bp marker. Lane 2: Male liver +ve control; Lane 3: Female granulosa cell +ve control. Lanes 4 to 19: cleaved embryos. Lane 19: reagent control (RC).

Table S7. Immunoblotting blotting for BHMT. Tissue/cell quantities and lysis buffer volumes (A), antibodies and antibody concentrations (B).

**A. Tissue/cells**	**Quantity**	**Lysis buffer (µL)**
Liver	20 mg	500
Granulosa cells:		
Rat	$1x10^6$ cells	50
Bovine, ovine, porcine	3 antral follicles	200
Theca cells:		
Bovine, ovine, porcine	3 antral follicles	200
HepG2 cells	$1x10^6$ cells	100
KGN cells	$1x10^6$ cells	100

**B. Antibodies**	**BHMT**	**Histone H3**
Primary	Anti-BHMT (1 mg/mL, rabbit polyclonal) (GTX102983, Acris antibodies)	Anti-Histone H3 (0.5 mg/mL, rabbit polyclonal) (ab 1791, Abcam)
All species	1:5000<br>1:50	1:100,000
Secondary	Horseradish Peroxidae (HRP)-conjugated goat anti-rabbit IgG (A9169, Sigma Aldrich)	
All species	1:30,000	1:100,000
Predicted size	45 kDa	15 kDa

Table S8. Summary of Bismark Final Alignment report. Total sequence pairs read following quality trimming[a]. Number of paired-alignments with unique best hit[b]. Mapping efficiency (%) is a measure of the sequence pairs that map uniquely to the reference genome[c].

**Lineage**	**ICM**		**TE**	
**Methionine (µmol/L)**	**50**	**10**	**50**	**10**
**Replicate**				
**1**	64,582,554[a] 18,826,935[b] (29.2)[c]	70,125,142[a] 20,930,071[b] (29.8)[c]	83,302,871[a] 23,538,817[b] (28.3)[c]	102,310,417[a] 31,671,654[b] (31.0)[c]
**2**	63,884,223[a] 18,968,032[b] (29.7)[c]	82,902,050[a] 25,559,151[b] (30.8)[c]	77,140,135[a] 21,008,778[b] (27.2)[c]	83,057,058[a] 24,657,595[b] (29.7)[c]
**3**	89,320,723[a] 26,120,363[b] (29.2)[c]	78,350,625[a] 22,750,802[b] (29.0)[c]	59,062,149[a] 17,074,286[b] (28.9)[c]	81,772,406[a] 23,879,134[b] (29.2)[c]
